# Contrasting gene expression patterns in grain of high and low asparagine wheat genotypes in response to sulphur supply

**DOI:** 10.1186/s12864-019-5991-8

**Published:** 2019-08-01

**Authors:** Tanya Y. Curtis, Sarah Raffan, Yongfang Wan, Robert King, Asier Gonzalez-Uriarte, Nigel G. Halford

**Affiliations:** 1 Plant Sciences Department, Rothamsted Research, Harpenden, Hertfordshire AL5 2JQ UK; 2 Computational and Analytical Sciences Department, Rothamsted Research, Harpenden, Hertfordshire AL5 2JQ UK; 3 Present Address: Curtis Analytics Ltd, Daniel Hall Building, Rothamsted RoCRE, Harpenden, AL5 2JQ UK; 4 Present Address: The European Bioinformatics Institute (EMBL-EBI), Wellcome Genome Campus, Hinxton, Cambridgeshire, CB10 1SD UK

**Keywords:** Asparagine synthetase, Amino acid metabolism, Acrylamide, bZIP, Crop composition, Food safety, RNA-seq, Sulphur, *Triticum aestivum*, Wheat

## Abstract

**Background:**

Free asparagine is the precursor for acrylamide formation during cooking and processing of grains, tubers, beans and other crop products. In wheat grain, free asparagine, free glutamine and total free amino acids accumulate to high levels in response to sulphur deficiency. In this study, RNA-seq data were acquired for the embryo and endosperm of two genotypes of bread wheat, Spark and SR3, growing under conditions of sulphur sufficiency and deficiency, and sampled at 14 and 21 days post anthesis (dpa). The aim was to provide new knowledge and understanding of the genetic control of asparagine accumulation and breakdown in wheat grain.

**Results:**

There were clear differences in gene expression patterns between the genotypes. Sulphur responses were greater at 21 dpa than 14 dpa, and more evident in SR3 than Spark. *TaASN2* was the most highly expressed asparagine synthetase gene in the grain, with expression in the embryo much higher than in the endosperm, and higher in Spark than SR3 during early development. There was a trend for genes encoding enzymes of nitrogen assimilation to be more highly expressed in Spark than SR3 when sulphur was supplied. *TaASN2* expression in the embryo of SR3 increased in response to sulphur deficiency at 21 dpa, although this was not observed in Spark. This increase in *TaASN2* expression was accompanied by an increase in glutamine synthetase gene expression and a decrease in asparaginase gene expression. Asparagine synthetase and asparaginase gene expression in the endosperm responded in the opposite way. Genes encoding regulatory protein kinases, SnRK1 and GCN2, both implicated in regulating asparagine synthetase gene expression, also responded to sulphur deficiency. Genes encoding bZIP transcription factors, including Opaque2/bZIP9, SPA/bZIP25 and BLZ1/OHP1/bZIP63, all of which contain SnRK1 target sites, were also expressed. Homeologues of many genes showed differential expression patterns and responses, including *TaASN2*.

**Conclusions:**

Data on the genetic control of free asparagine accumulation in wheat grain and its response to sulphur supply showed grain asparagine levels to be determined in the embryo, and identified genes encoding signalling and metabolic proteins involved in asparagine metabolism that respond to sulphur availability.

**Electronic supplementary material:**

The online version of this article (10.1186/s12864-019-5991-8) contains supplementary material, which is available to authorized users.

## Background

Interest in the synthesis, accumulation and breakdown of asparagine in crop plants has been reinvigorated in recent years due to the discovery that free (soluble, non-protein) asparagine is the precursor for acrylamide formation during cooking and processing [1–3] and its concentration is the main determinant of acrylamide-forming potential in wheat and other cereals [4–8]. Acrylamide forms in the Maillard reaction, which also requires reducing sugars, such as glucose, fructose and maltose, but the carbon skeleton of the acrylamide that forms is derived from free asparagine.

Fried, baked, roasted and toasted cereal, coffee and potato products are the main sources of dietary acrylamide. Acrylamide is classed as a Group 2a human carcinogen [9] and in 2015 the European Food Safety Authority (EFSA) Expert Panel on Contaminants in the Food Chain (CONTAM) issued a report concluding that the margins of exposure to dietary acrylamide indicated ‘a concern for neoplastic effects’ [10]. Subsequently (April 2018), Commission Regulation (EU) 2017/2158 came into force across the European Union, introducing compulsory risk management measures that apply to all food businesses [11].

The development of crop varieties with reduced acrylamide-forming potential may enable the food industry to comply with regulations without costly changes to manufacturing lines or reduced product quality. In the case of cereals, this means varieties with reduced and more consistent free asparagine concentration in the grain. There is, therefore, a need for greater knowledge and understanding of the genetic control of asparagine synthesis, accumulation and breakdown.

**Fig. 1:**
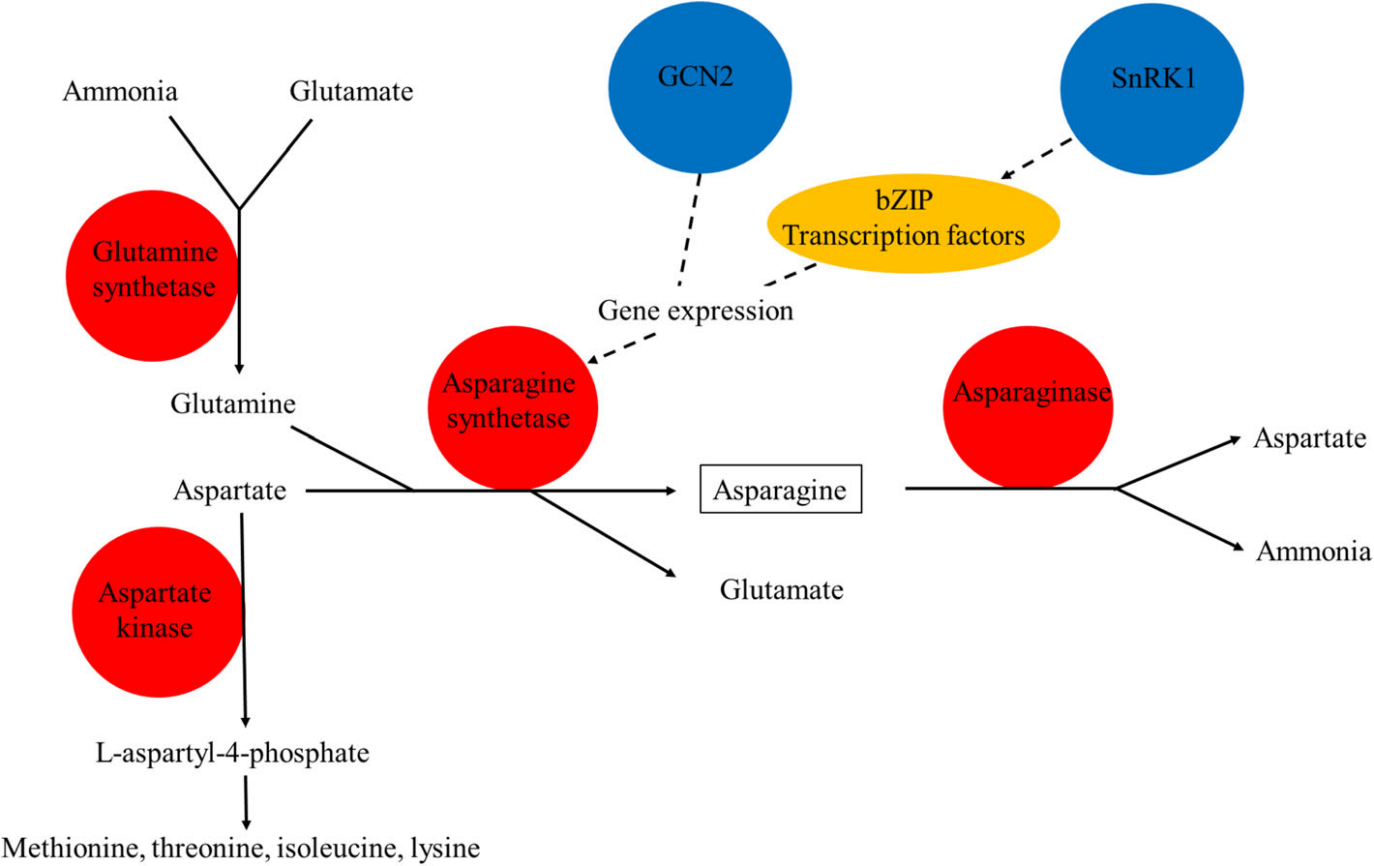
Diagram representing the metabolic enzymes (red circles), regulatory protein kinases (blue circles) and transcription factors (yellow oblong) at the heart of the asparagine metabolism network. A much more extended network has been constructed by Curtis et al. [12]

Asparagine is synthesised through the ATP-dependent transfer of the amino group of glutamine to a molecule of aspartate to generate glutamate and asparagine, a reaction catalysed by the enzyme asparagine synthetase. An extensive network comprising genes, enzymes, transcription factors and regulatory proteins has been constructed [12] and the components of the core of that network are shown in Fig. 1. Free asparagine accumulates in many plant tissues in response to a range of abiotic and biotic stresses, as well as during normal physiological processes such as seed germination [13]. In wheat grain, it accumulates to very high levels in response to sulphur deficiency [4–7] and poor disease control [14]. Sulphur deficiency also brings about large increases in free glutamine and total free amino acid concentrations [6, 15]. There are also substantial differences in the free asparagine concentration of grain from different wheat varieties and genotypes [15].

In the present study, RNA-seq analysis was used to compare two wheat genotypes, variety Spark and a doubled haploid line, SR3, from a Spark × Rialto mapping population [16]. SR3 has previously been shown to have a lower concentration of free asparagine in the grain than Spark (1.68 versus 2.71 mmol per kg when grown in compost, a difference of 61% with respect to the lower figure, and 2.05 versus 2.54 mmol per kg when grown in vermiculite, a difference of 24% with respect to the lower figure [6]). It also has a lower concentration of total free amino acids, particularly under conditions of sulphur deficiency [6]. The two genotypes were grown under conditions of sulphur sufficiency and deficiency, and the data analysed to identify the genes involved in the response to sulphur deficiency and how the sulphur response differed between the two genotypes, focussing on genes involved in asparagine metabolism and its regulation, and nitrogen assimilation.

## Results

Winter wheat genotypes SR3 and Spark were grown in a glasshouse with and without sulphur supplied and grain samples taken at 14 and 21 days post-anthesis (dpa). The embryo and endosperm were separated, and four embryo and four endosperm samples (biological replicates) were analysed for each genotype, time-point and treatment, making a total of 64 samples from which RNA was prepared for RNA-seq analysis. One sample for each of the genotypes was discarded because the RNA was not of adequate quality.

### Exploratory analyses

**Fig. 2:**
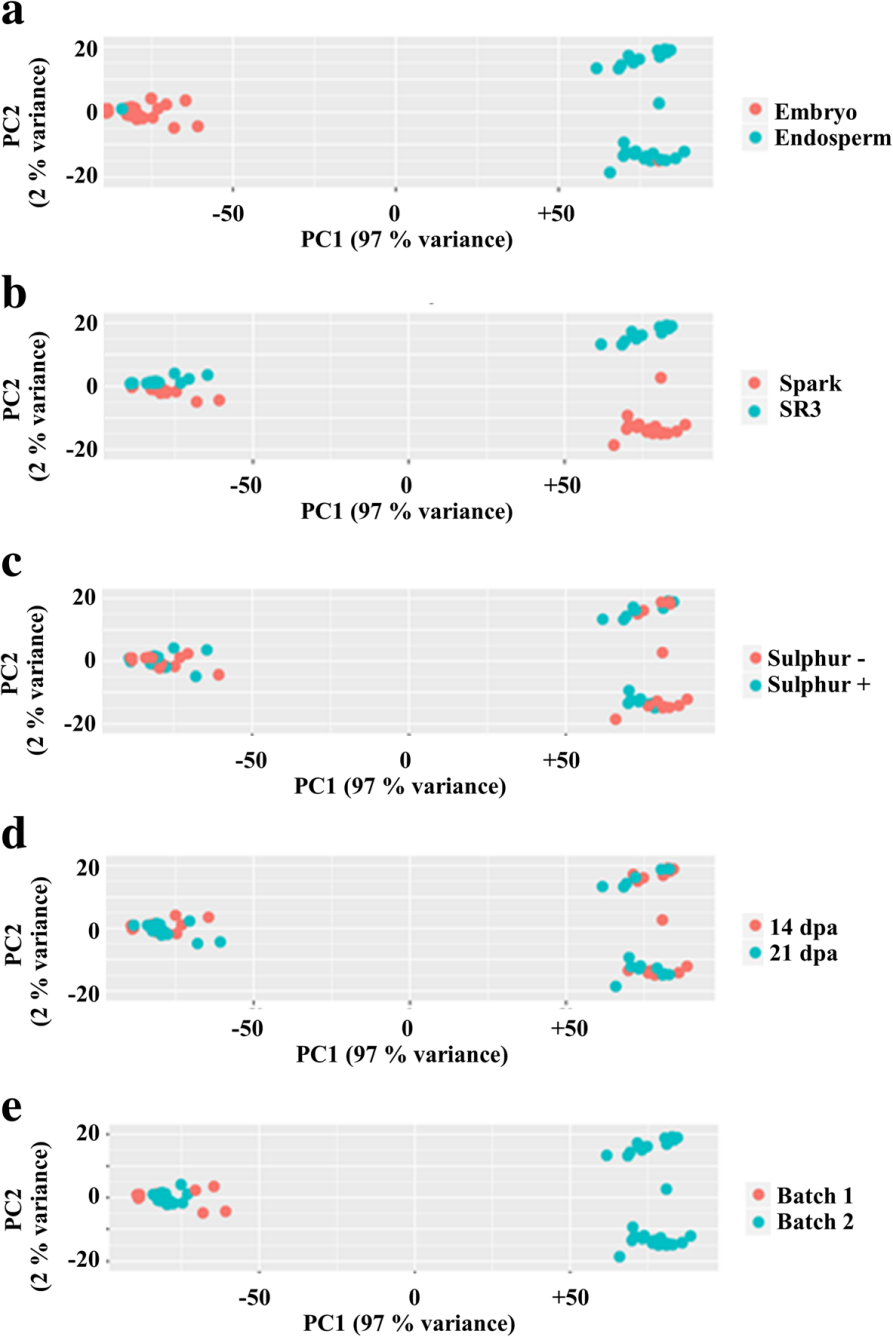
Plots derived from Principal Component analysis (PCA) of the data showing the variance due to: **a** Tissue. **b** Genotype. **c** Sulphur treatment. **d** Timepoint. **e** Batch

A principal component analysis (PCA) was performed and the resulting plots are shown in Fig. 2. The PCA showed the main source of variation to be tissue (Fig. 2a), as would be expected. This plot identified two samples that seemed to have been mislabelled, and these were excluded from further analysis. The secondary source of variation was the genotype (Fig. 2b), followed by treatment (S+ versus S-; Fig. 2c) and time (14 dpa versus 21 dpa (Fig. 2d). In addition, the analysis showed one batch of data to be slightly different from a second (Fig. 2e). Three further samples were dropped due to poor clustering, reducing the number of samples to 57. The raw data have been deposited in European Nucleotide Archive (ENA) and is publicly available (https://www.ebi.ac.uk/ena) using the study accession: PRJEB31122.

The data were analysed using the DESeq2 r package to make pairwise comparisons of high versus low sulphur for each of the eight genotype × timepoint × treatment combinations. Genes were classified as differentially expressed using an adjusted *p*-value of < 0.05. Comparisons were made between high and low sulphur treatments in the two genotypes, at different stages and in the two tissue types (Table 1A). Overall, more genes showed differential expression in response to the treatment in SR3 than Spark, and there was a much greater response by 21 dpa than 14 dpa. There were also more genes changing in the endosperm than the embryo, particularly in Spark (only two genes showed differential expression in Spark embryos, even at 21 dpa). Table 1B contrasts the responses in the embryo and endosperm and shows that a very different set of genes changed in the two tissues, with only a relatively small number of genes responding in both. Overall, the numbers of genes responding to sulphur were much higher than those reported by Yu et al. [17].

**Table 1:** Summary of the changes in gene expression in response to sulphur. A. The number of genes that showed significant (*p* < 0.05) changes in expression in response to sulphur deficiency, in total and split into upregulated and downregulated. B. As for A, but contrasting the gene expression changes between tissue types

A												
	21 days						14 days					
	Endosperm			Embryo			Endosperm			Embryo		
	Total	Up	Down	Total	Up	Down	Total	Up	Down	Total	Up	Down
Spark Total	5151	2422	2729	2	2	0	1	0	1	0	0	0
SR3 Total	8905	3179	5726	5223	1901	3322	869	344	525	646	633	13
In both (Spark)	1707	712	995	0	0	0	0	0	0	0	0	0
In both (SR3)	1707	659	1048	0	0	0	0	0	0	0	0	0
Spark only	3444	1710	1734	2	0	2	1	0	1	0	0	0
SR3 only	7198	2520	4678	5223	1901	3322	869	344	525	646	633	13
B												
	21 days						14 days					
	Spark			SR3			Spark			SR3		
	Total	Up	Down	Total	Up	Down	Total	Up	Down	Total	Up	Down
Endosperm	5151	2422	2729	8905	3179	5726	1	0	1	869	344	525
Embryo	2	2	0	5223	1901	3322	0	0	0	646	330	316
In both (end)	1	1	0	951	322	629	0	0	0	27	27	0
In both (emb)	1	1	0	951	421	530	0	0	0	27	26	1
Endosperm only	5150	2421	2729	7954	2857	5097	1	0	1	842	462	380
Embryo only	1	1	0	4272	1480	2792	0	0	0	619	607	12

Functional enrichment analysis was performed separately for the differentially expressed gene lists. Three of the pairwise comparisons (variety Spark, endosperm, 14 dpa; embryo, 14 and 21 dpa) did not show enrichment because of the small number of differentially expressed genes. The results of the enrichment of the rest of the comparisons are given in Additional file 1.

### Confirmatory analyses

**Fig. 3:**
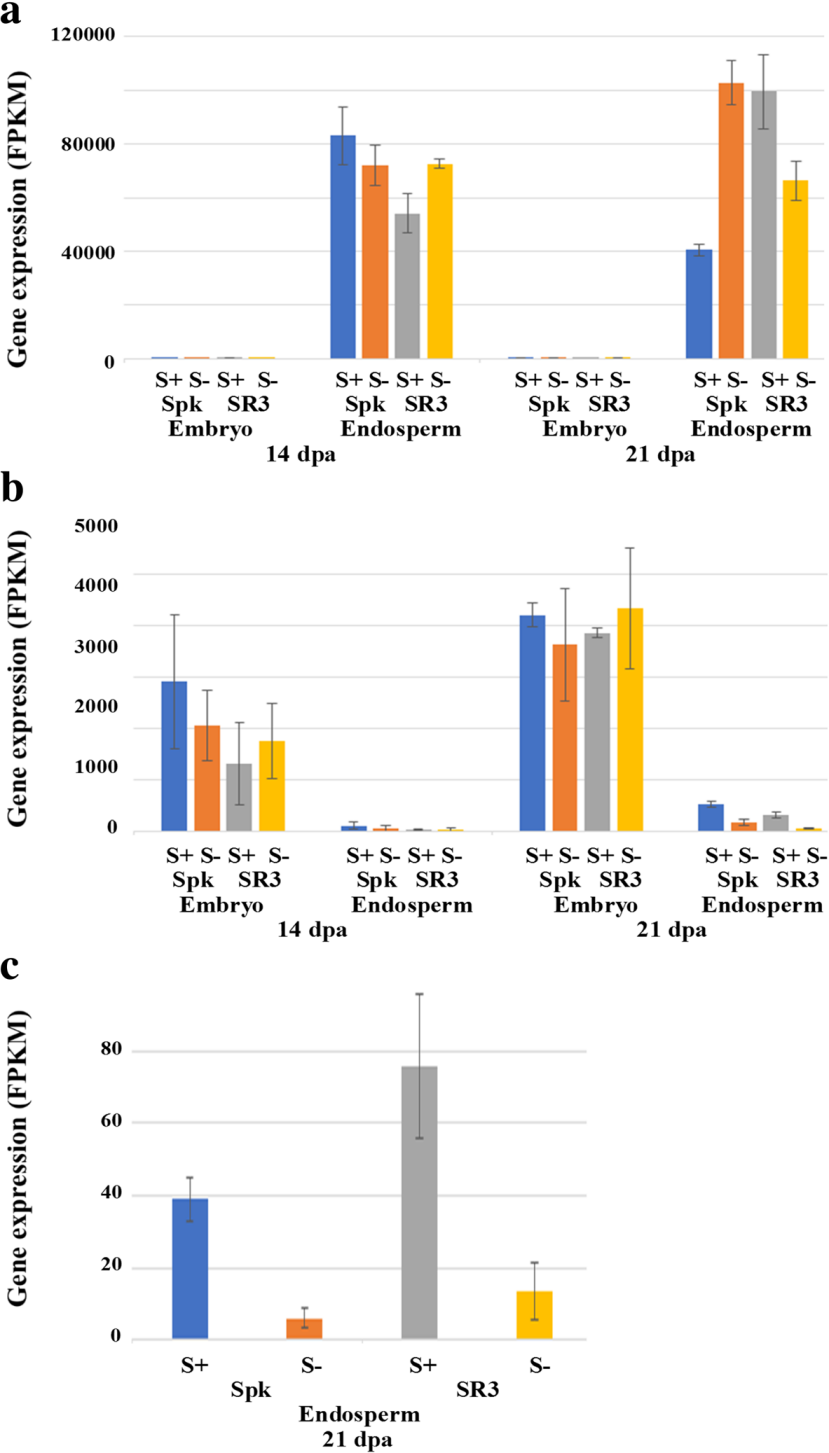
Expression patterns of well-characterised genes in the embryo and endosperm of developing grain from two wheat (*Triticum aestivum*) genotypes, Spark and SR3, to validate the RNAseq dataset. The plants were grown with sulphur either supplied (S+) or withheld (S-) and sampled at 14 and 21 days post-anthesis (dpa). **a** Endosperm-specific expression of low molecular weight glutenin subunit gene. **b** Embryo-specific expression of late embryogenesis abundant (*LEA12*) gene. **c** Sulphur response of ethylene-insensitive 3-like 5 (*EIL5*) gene. Gene expression is represented in fragments per kilobase of transcript per million mapped reads (FPKM). Gene reference numbers are given in Additional file 2

The validity of the data with respect to tissue type was confirmed by checking the expression of genes encoding prolamin storage proteins, which are known to be expressed endosperm-specifically [18]. These all showed clear, endosperm-specific expression, and this is shown graphically for a gene encoding a low molecular weight glutenin subunit in Fig. 3a. In contrast, the late embryogenesis abundant 12 (*LEA12*) gene was expressed at much higher levels in the embryo than the endosperm, as is typical of this class of genes (see [19] for review) (Fig. 3b). The relatively very high expression of the low molecular weight glutenin subunit gene is also in line with expectations [18].

Several genes are known to be responsive to sulphur feeding in wheat, but this has usually been demonstrated in seedlings rather than grain tissues [20]. Nevertheless, there were examples in the data of genes responding clearly to sulphur availability in the expected manner, and this is shown for an ethylene-insensitive 3-like 5 (*EIL5*) gene [21] in Fig. 3c. This gene was clearly induced by sulphur feeding in the endosperm at 21 dpa, although the response was not significant in the embryo at 21 dpa and was not apparent in either tissue at 14 dpa, showing the sulphur response to be much more evident at 21 dpa than 14 dpa. The reference numbers for these three genes are provided in Additional file 2.

### Genes of interest in asparagine synthesis, accumulation and breakdown

The aim of the study was to gain an understanding of the genetic factors responsible for the difference in free asparagine and total free amino acid concentration in the grain of Spark and SR3, and the factors involved in changes in free asparagine and total free amino acid accumulation in response to sulphur availability. The data on genes included in the network identified by Curtis et al. [12] as being involved in asparagine synthesis, turnover and accumulation were therefore analysed in detail.

### Asparagine synthetases

Wheat (*Triticum aestivum*) contains four asparagine synthetase genes, called *TaASN1*-*TaASN4* [22]. The enzymes encoded by these genes are very similar to each other, with molecular masses between 65 and 67 kDa. Heterologous expression and biochemical characterisation of TaASN1 and TaASN2 enzymes also showed them to have similar biochemical properties [23]. *TaASN1*, *TaASN2* and *TaASN4* are all single copy genes, located on chromosomes 5, 3 and 4, respectively, of each genome (A, B and D), although some varieties lack a *TaASN2* gene in the B genome [23]. The *ASN1* and *ASN2* genes of durum pasta wheat (*Triticum turgidum ssp. durum*) are also located on chromosomes 5 and 3, respectively [24]. Two copies of *TaASN3, TaASN3.1* and *TaASN3.2,* are present on chromosome 1 of each genome. *TaASN3.2* is annotated as *TaASN5* in some databases, but the similarities in gene structure and chromosomal location of *TaASN3.1* and *TaASN3.2* justify regarding them as two copies of the same gene. Gao et al. [22] examined the expression of *TaASN1*, *TaASN2* and *TaASN3* and showed *TaASN2* to be the most highly expressed in the grain. However, *TaASN1* was the most responsive to nitrogen availability and sulphur deficiency and had previously been shown to respond to salt stress, osmotic stress and ABA [25]. The *ASN1* gene of durum wheat has also been shown to respond to nitrogen availability [24].

**Fig. 4:**
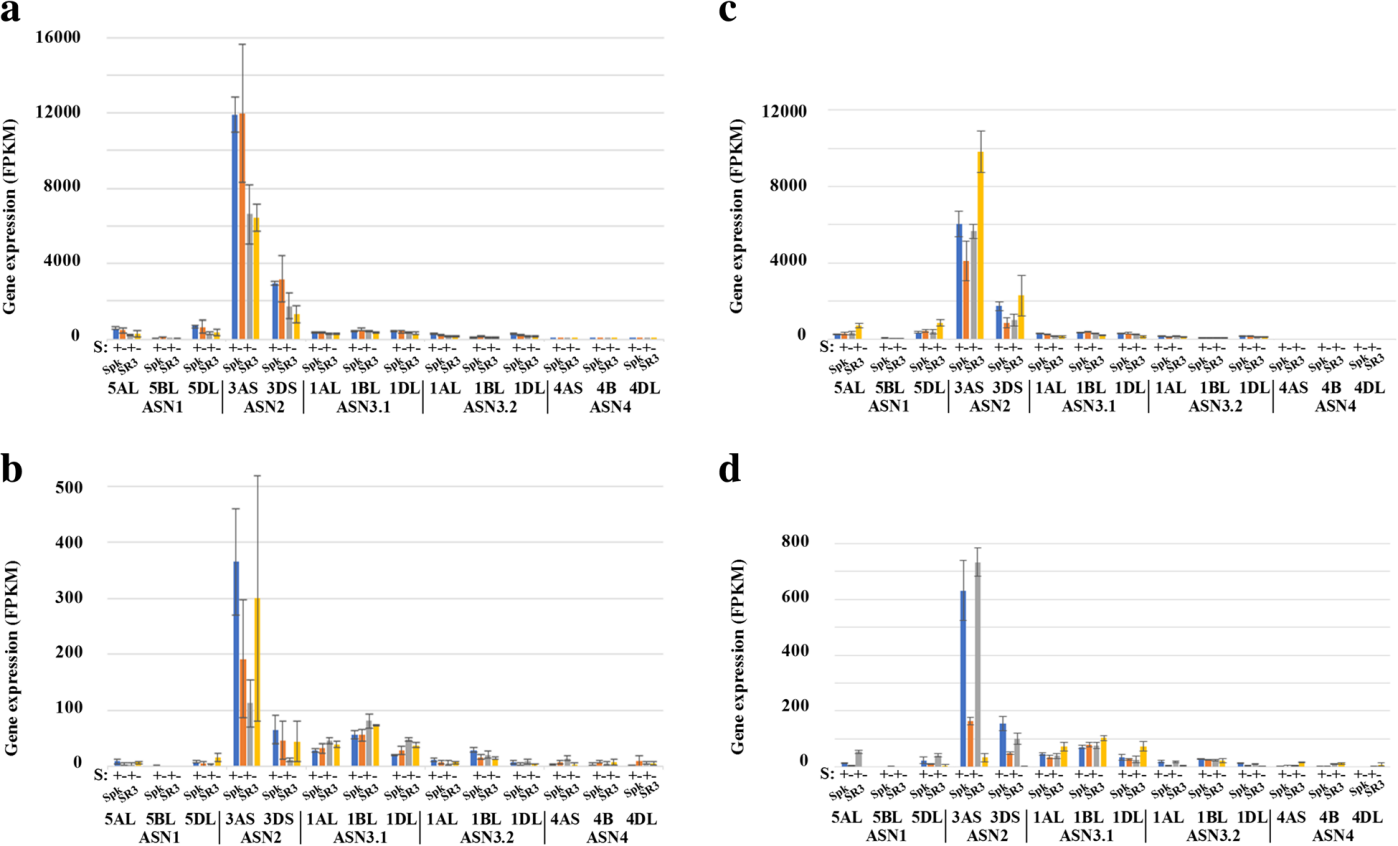
Expression levels (FPKM) of asparagine synthetase genes *TaASN1*, *TaASN2*, *TaASN3.1*, *TaASN3.2* and *TaASN4*, in the embryo and endosperm of developing grain from two wheat (*Triticum aestivum*) genotypes, Spark and SR3. The plants were grown with sulphur either supplied (+) or withheld (−). Results for each homeologue are shown separately, as indicated. **a** Embryo, 14 dpa. **b** Endosperm, 14 dpa. **c** Embryo, 21 dpa. **d** Endosperm, 21 dpa. Gene reference numbers are given in Additional file 2. The increase in expression of the *TaASN1* homeologue on chromosome 5D in response to sulphur deficiency in SR3 embryo at 21 dpa was significant (*p* = 0.0478), as was the increase in expression of both *TaASN2* homeologues (*p* = 0.038 and 0.047 for the 3A and 3D homeologues, respectively). The reduction of expression of the two *TaASN2* homeologues at 21 dpa in the endosperm in response to sulphur deficiency was also significant (*p* < 0.001 for both), as was the increase in expression of the 5A and 5D *TaASN1* homeologues (*p* < 0.001 and *p* = 0.0012, respectively)

Figure 4 shows the expression of all the *TaASN* genes in the present study, comparing the expression in Spark with SR3 in the embryo and endosperm for each homeologue separately, with and without sulphur supplied, at 14 dpa (Fig. 4a and b) and 21 dpa (Fig. 4c and d). The reference numbers for the genes are provided in Additional file 2. The data revealed much higher total asparagine synthetase gene expression in the embryo than in the endosperm (> 10-fold difference), consistent with previous findings [22]. *TaASN2* was the most highly expressed in both tissues, in both conditions (S+ and S-), and at both time-points, accounting for several times the expression of all the others combined, again consistent with previous findings [22]. This was despite the apparent lack of expression of a B genome *TaASN2* homeologue. Some varieties lack a B genome *TaASN2* homeologue [23], including Chinese Spring, which was the reference genome used in the study. Analysis of the RNA-seq reads did not reveal any single nucleotide polymorphisms to suggest that a B genome homeologue was being expressed; in other words, all the reads could be assigned to the A or D genome homeologues. We conclude that the B genome homeologue is absent in SR3 and Spark, as it is in Chinese Spring, or is present but not expressed. The data also showed for the first time the A genome homeologue of *TaASN2* to be much more highly expressed (> 3-fold difference) than the D genome homeologue. This meant that the A genome homeologue of *TaASN2* was responsible for more than half of the total asparagine synthetase gene expression in the grain under both treatments and at both timepoints.

There were also differences in expression of the three *TaASN1* homeologues, with the B genome homeologue expressed at lower levels than the A and D genome homeologues. Of the two *TaASN3* genes, *TaASN3.1* was much more highly expressed than *TaASN3.2*, while *TaASN4* expression was very low in all the samples.

There was approximately 80% more asparagine synthetase gene expression in the embryo of Spark compared with SR3 at 14 dpa, under both sulphur sufficiency and deficiency, almost entirely due to higher levels of *TaASN2* expression in Spark compared with SR3 at this time-point (Fig. 4a). This could explain the higher concentration of free asparagine in Spark grain compared with SR3 [6]. The difference had reduced to approximately 15% by 21 dpa, under sulphur sufficiency (Fig. 4c), while under sulphur deficiency at this point expression was actually higher in SR3 than in Spark.

The effects of sulphur were complex and differed between the genotypes. There was no effect of sulphur in the embryo of either genotype at 14 dpa (Fig. 4a), but at 21 dpa the expression of the *TaASN1* homeologue on chromosome 5D increased in response to sulphur deficiency in SR3 (*p* = 0.0478), as did the expression of both *TaASN2* homeologues (*p* = 0.038 and 0.047, for the homeologues on chromosomes 3A and 3D, respectively) (Fig. 4c). Clearly, such a response could explain the increased free asparagine accumulation observed in wheat grain in response to sulphur deficiency. However, the same response was not observed in Spark, in which both *TaASN1* and *TaASN2* expression decreased rather than increased in response to the low sulphur treatment. Given that the response seen in SR3 only occurred at the later timepoint of 21 dpa, one possible explanation is that Spark was just behind SR3 developmentally and it was too early to see the response, but this is speculative.

In the endosperm at 14 dpa (Fig. 4b), sulphur deficiency reduced expression of both *TaASN1* and *TaASN2* in Spark but increased it in SR3, although levels of expression were still only a fraction of those seen in the embryo. There was no great effect of sulphur deficiency on *TaASN3* expression, and while *TaASN4* expression increased in Spark but decreased in SR3, this was at comparatively very low levels of expression. At 21 dpa, sulphur deficiency reduced *TaASN2* expression in both genotypes (*p* < 0.001 for both homeologues) but increased *TaASN1* expression (*p* < 0.001 and *p* = 0.0012, respectively, for the homeologues on 5A and 5D; change not significant (*p* > 0.05) for the 5B homeologue). *TaASN1* therefore responded to sulphur in this tissue in the same way as in leaves. However, in contrast to the situation in leaves [22], its expression was dwarfed by that of *TaASN2*.

### Asparaginases

**Fig. 5:**
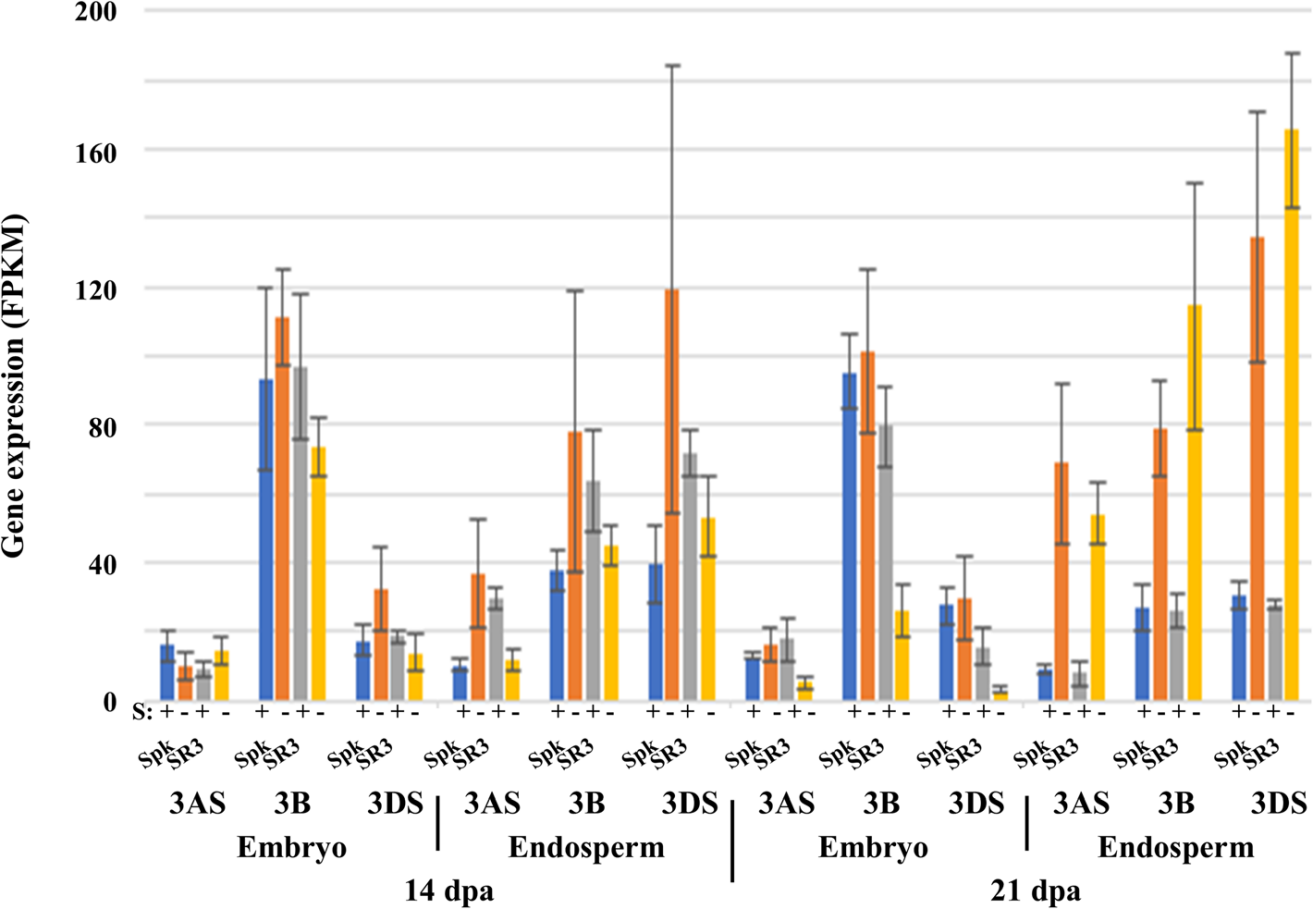
Expression levels (FPKM) of asparaginase genes in the embryo and endosperm of developing grain from wheat (*Triticum aestivum*) genotypes Spark and SR3. Plants were grown with sulphur either supplied (+) or withheld (−) and analysed at 14 and 21 dpa. Results for each homeologue are shown separately, as indicated. Gene reference numbers are given in Additional file 2. The increase in expression of all three homeologues in response to sulphur deficiency at 21 dpa in the endosperm of both Spark and SR3 was significant (*p* < 0.01), as was the decrease in expression of the B genome homeologue in the embryo in response to sulphur deficiency at 21 dpa in SR3 (*p* = 0.017)

Seven putative asparaginase genes were identified on each genome, based on the derived amino acid sequences of the encoded proteins (they are not annotated as asparaginases in the EnsemblPlants database). The reference numbers for the genes are given in Additional file 2. Six of the genes were located on chromosome 2A, with five homeologues in each case on 2B and 2D. Two unassigned sequences were probably the sixth 2B and 2D homeologues. The other gene was located on chromosome 3, with homeologues on each of 3AS, 3B and 3DS. This gene encoded a protein with 96% identity with an *Aegilops tauschii* asparaginase (accession number XP_020153640) and was of interest because all three homeologues showed significant (*p* < 0.01) increases in expression in response to sulphur deficiency at 21 dpa in the endosperm of both Spark and SR3 (Fig. 5). All three homeologues were also expressed in the embryo, but the B genome version was expressed much more highly than the other two (Fig. 5). All three decreased in expression in the embryo in response to sulphur deficiency at 21 dpa in SR3, the reduction in expression of the B genome homeologue being significant (*p* = 0.017). These responses meant that there was more asparaginase gene expression in the embryo than in the endosperm under sulphur sufficiency, but this was reversed under sulphur deficiency, particularly at 21 dpa.

### Aspartate kinase

Aspartate kinase has the potential to compete with asparagine synthetase for aspartate (Fig. 1). Genes encoding the enzyme were identified on chromosome 3 (3AL, 3B and 3DL), 4 (4AL only), and 5 (5BL and two homeologues on 5DL). Expression of these genes was generally higher in the embryo than the endosperm, with little change from 14 to 21 dpa and little difference between the genotypes (Additional file 2: Figure S1). There were some responses to sulphur, notably of one of the 5DL homeologues in the endosperm at 21 dpa, but they were not significant (*p* > 0.05). Overall the data did not suggest a major role for aspartate kinase in regulating free asparagine levels.

### Enzymes of nitrogen assimilation: glutamine synthetase, glutamate synthase (GOGAT), nitrate reductase and nitrite reductase

Free asparagine becomes the most abundant free amino acid in wheat grain in response to sulphur deficiency, but there are also large increases in free glutamine and total free amino acid concentrations [4–7]. This makes enzymes of nitrogen assimilation of interest and the data showed that genes encoding nitrate reductase, nitrite reductase, glutamine synthetase and glutamate synthase (glutamine 2-oxyoglutarate aminotransferase; GOGAT) were all expressed.

**Fig. 6:**
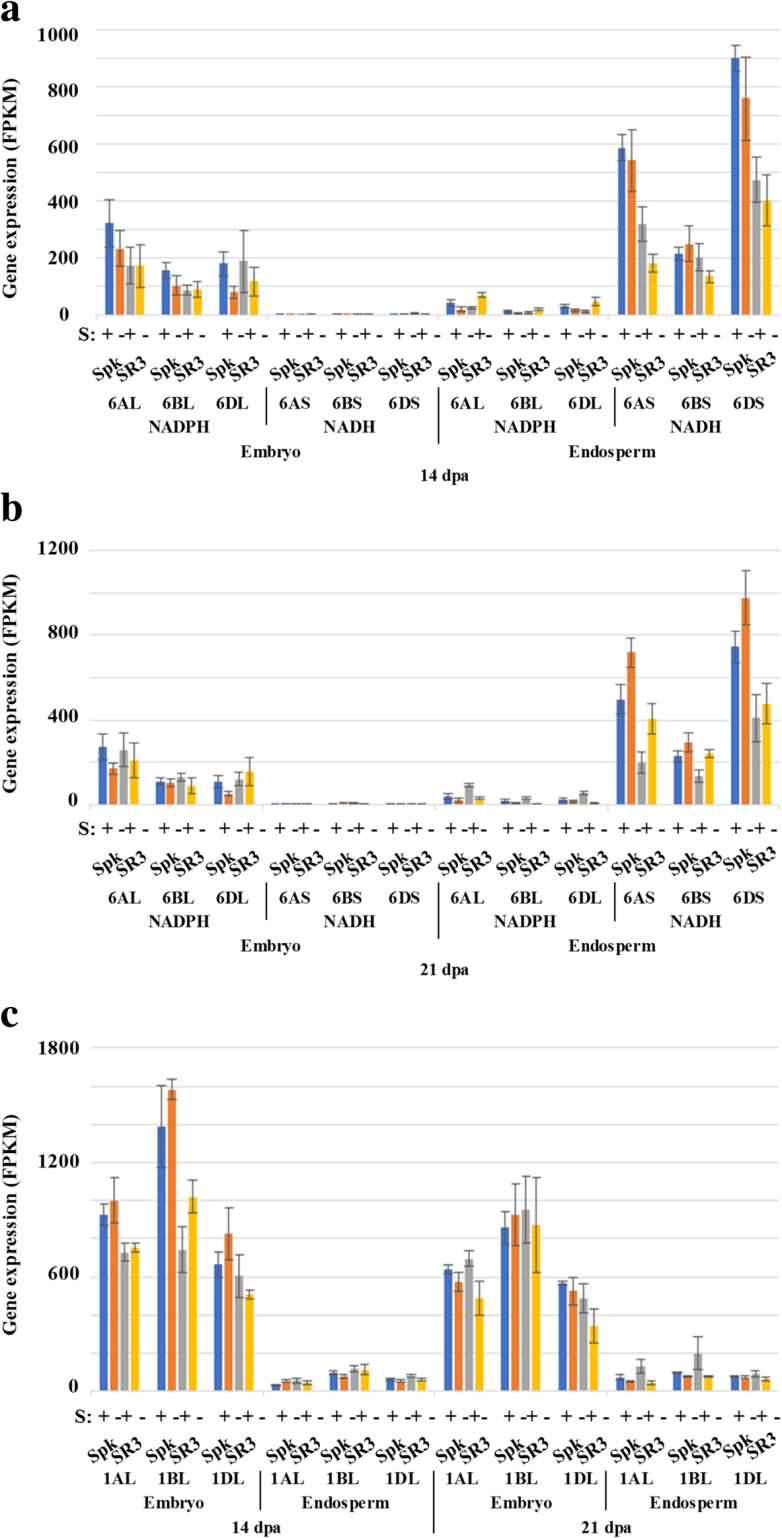
Expression levels (FPKM) of nitrate and nitrite reductase genes in the embryo and endosperm of developing grain from wheat (*Triticum aestivum*) genotypes Spark and SR3. Plants were grown with sulphur either supplied (+) or withheld (−) and analysed at 14 and 21 dpa. **a** and **b** Genes encoding NADH- and NADPH-dependent nitrate reductase at 14 and 21 dpa, respectively. **c** Gene encoding nitrite reductase. Results for each homeologue are shown separately, as indicated. Gene reference numbers are given in Additional file 2. The decrease in expression of the NADPH-dependent nitrate reductase gene in response to sulphur deficiency in the endosperm of SR3 at 21 dpa was significant (*p* < 0.001)

The data for nitrate reductase and nitrite reductase are shown graphically in Fig. 6. The fact that these genes were expressed suggests strongly that nitrate was being transported into the grain, rather than nitrogen being imported entirely in the form of amino acids or other organic compounds. An NADPH-dependent nitrate reductase-encoding gene was identified on chromosome 6, with homeologues on 6AL, 6BL and 6DL, all of which were expressed much more highly in the embryo than in the endosperm (Fig. 6a and b). Conversely, a gene encoding NADH-dependent nitrate reductase was also identified on chromosome 6, with homeologues on 6AS, 6BS and 6DS, and all of these were expressed much more highly in the endosperm than the embryo. There was a trend for expression of the gene encoding NADH-dependent nitrate reductase to increase in response to sulphur deficiency in the endosperm at 21 dpa but it was not significant (*p* > 0.05) (Fig. 6b). It was also notable that expression of the homeologues on chromosomes 6AS, 6DS, 6AL and 6BL was higher in Spark than SR3 at 14 dpa, with expression of the 6AS and 6DS homeologues continuing to be higher at 21 dpa. All three homeologues encoding the NADPH-dependent nitrate reductase decreased in expression in the endosperm of SR3 in response to sulphur deficiency (*p* < 0.001), albeit from a low level.

A nitrite reductase gene was identified on chromosome 1, with homeologues on 1AL, 1BL and 1DL. These were expressed in both tissues but much more highly in the embryo than the endosperm (Fig. 6c). At 14 dpa, expression of the 1AL and 1BL homeologues in the embryo was much higher in Spark than SR3. A gene was also identified on chromosome 6, but this was expressed at much lower levels in both tissues.

**Fig. 7:**
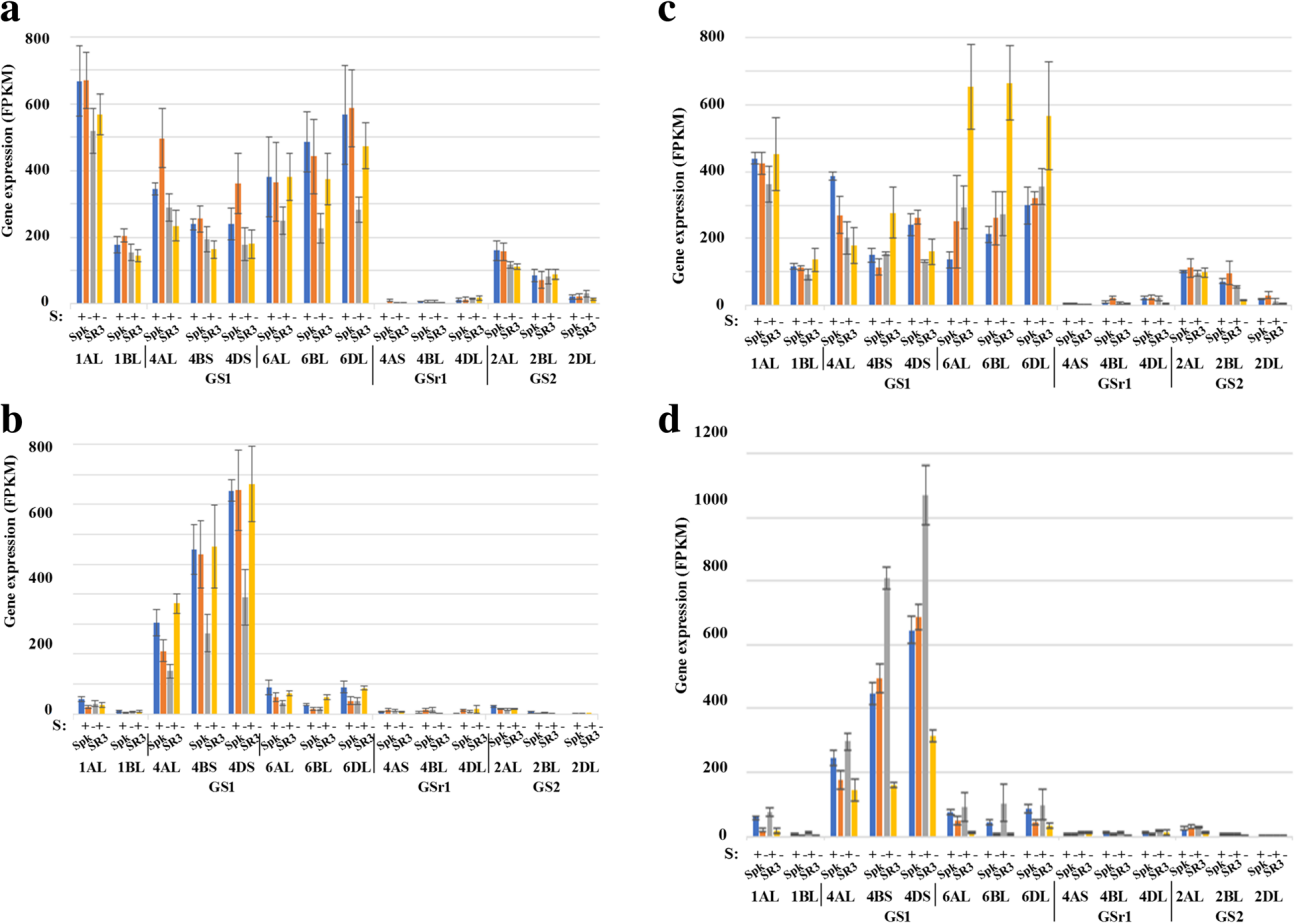
Expression levels (FPKM) of genes encoding glutamine synthetases GS1, GSr1 and GS2 in the embryo and endosperm of developing grain from wheat (*Triticum aestivum*) genotypes Spark and SR3. The plants were grown with sulphur either supplied (+) or withheld (−). Results for each homeologue are shown separately, as indicated. **a** Embryo, 14 dpa. **b** Endosperm, 14 dpa. **c** Embryo, 21 dpa. **d** Endosperm, 21 dpa. Gene reference numbers are given in Additional file 2. The increase in expression of the 4AL and 6BL homeologues in the endosperm in SR3 at 14 dpa in response to sulphur deficiency was significant (*p* = 0.00113 and 0.0299, respectively), as was the increase in expression of the 6AL and 6BL homeologues in the embryo of SR3 at 21 dpa (*p* = 0.0401 and 0.0164, respectively). In the endosperm at 21 dpa, the decrease in expression of the 6AL, 6BL, 6DL, 4DS, 4AL and 1AL genes in SR3 in response to sulphur deficiency was significant (*p* < 0.001 for 1AL, 6AL, 4BS and 4DS; *p* = 0.01961 for 6BL and 0.04599 for 6 DL)

Five genes encoding glutamine synthetase were identified, with GS1 (cytosolic) encoded by genes on chromosome 1, with homeologues on 1AL and 1BL (no 1D version), chromosome 4 (4AL, 4BS and 4DS) and chromosome 6 (6AL, 6BL and 6DL). GSr1 (cytosolic) was encoded by a gene on chromosome 4 (4AS, 4BL and 4DL), while GS2 (plastidic) was encoded by a gene on chromosome 2 (2AL, 2BL and 2DL). The genes encoding GSr1 and GS2 were expressed at relatively low levels (not surprisingly in the case of plastidic GS2) (Fig. 7). The GS1-encoding genes on chromosomes 1 and 6 were expressed much more highly in the embryo than the endosperm (Fig. 7a and c versus b and d), while the gene on chromosome 4 was expressed at high levels in both tissues. This meant that, overall, there was more expression of GS1 in the embryo than the endosperm. At 14 dpa under sulphur sufficiency, there was a trend for higher levels of expression in Spark than SR3 (Fig. 7a and b). This was most evident for the gene on chromosome 6 in the embryo and chromosome 4 in the endosperm. By 21 dpa this was reversed for some of the homeologues, and there were also contrasting responses to sulphur deficiency. There were large increases in expression of the 4AL and 6BL homeologues in the endosperm in SR3 at 14 dpa (Fig. 7b) (*p* = 0.00113 and 0.0299, respectively), albeit that the 6AL homeologue was from a relatively low level. At 21 dpa in the embryo there was no clear response to sulphur in Spark (Fig. 7c), whereas in SR3 the expression of the 6AL and 6BL homeologues increased in response to sulphur deficiency (*p* = 0.0401 and 0.0164, respectively). In the endosperm at 21 dpa, on the other hand, expression of the 1AL, 4AL, 4DS, 6AL, 6BL and 6DL genes decreased in SR3 in response to sulphur deficiency (Fig. 7d) (*p* < 0.001 for 1AL, 4BS, 4DS and 6AL; *p* = 0.01961 for 6BL and 0.04599 for 6DL). Some genes showed the same response in Spark, but the highly expressed gene on chromosomes 4 did not. Yu et al. [17] also reported that GS gene expression was repressed by sulphur, but that study analysed expression at 7 dpa and only looked at the gene on chromosome 6.

A single gene encoding glutamine oxoglutarate aminotransferase (GOGAT) was identified on chromosome 3 (3AL, 3BL and 3DL) (Additional file 2: Figure S2). The gene was expressed at much higher levels in the embryo than the endosperm. There was a trend for higher expression in the embryo under sulphur deficiency, but the differences were not significant (*p* > 0.05).

### Sucrose nonfermenting-1-related protein kinase-1 (SnRK1)

Sucrose nonfermenting-1-related protein kinase-1 (SnRK1) controls carbon metabolism through the modulation of enzyme activity and gene expression, including the re-allocation of carbon in response to a range of abiotic stresses (see [26, 27] for review). SnRK1 also promotes starch accumulation in potato tubers [28] and has been associated with the appearance of starch granules during rice endosperm development [29]. Its role in regulating asparagine synthetase gene expression has been demonstrated in Arabidopsis [30].

**Fig. 8:**
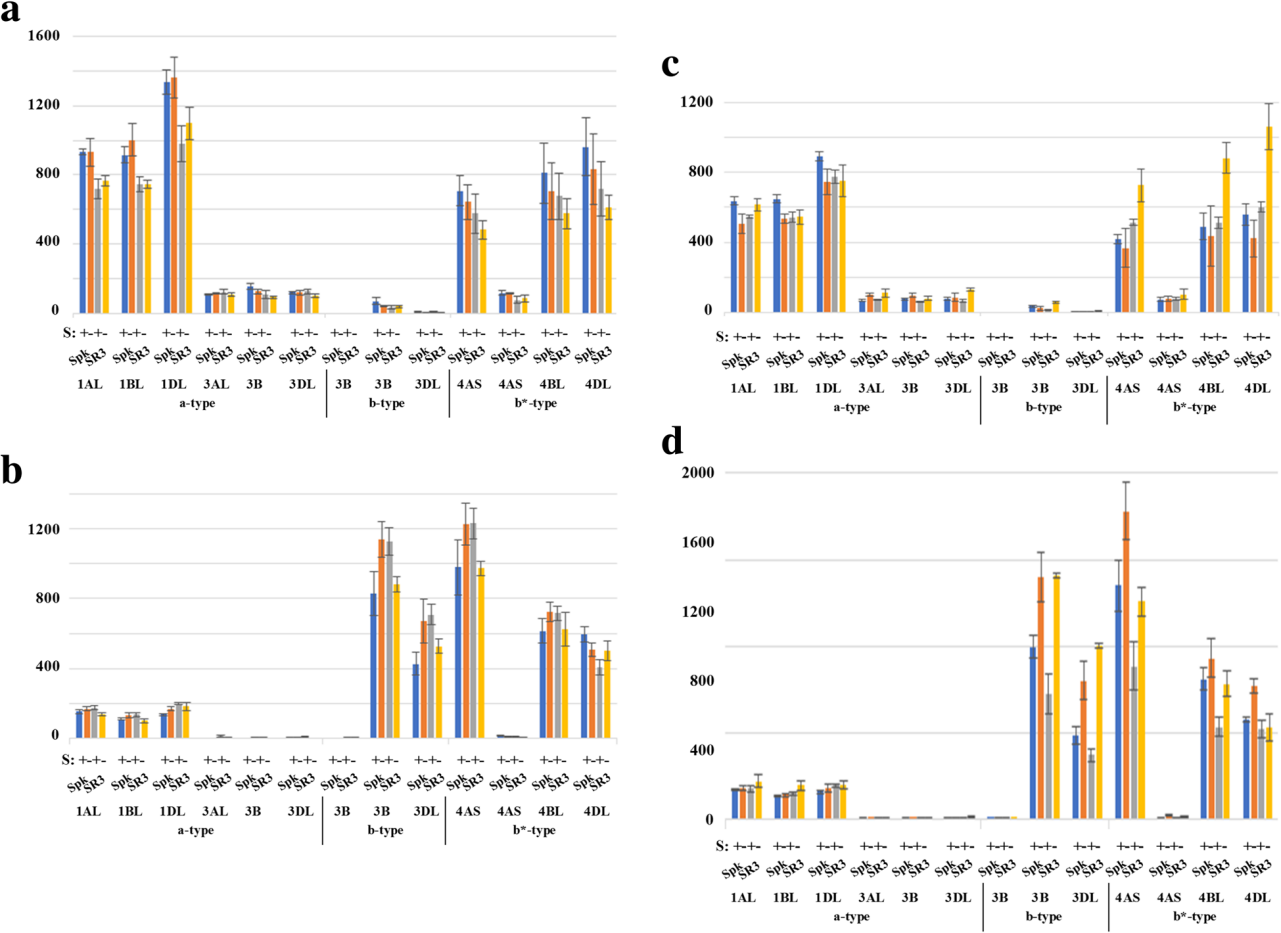
Expression levels (FPKM) of genes encoding SNF1-related protein kinase-1 (SnRK1) type a, b and b*, in the embryo and endosperm of developing grain from wheat (*Triticum aestivum*) genotypes Spark and SR3. The plants were grown with sulphur either supplied (+) or withheld (−). Results for each homeologue are shown separately, as indicated. **a** Embryo, 14 dpa. **b** Endosperm, 14 dpa. **c** Embryo, 21 dpa. **d** Endosperm, 21 dpa. Gene reference numbers are given in Additional file 2. The increase in expression of the *SnRK1b* homeologues on chromosomes 3B and 3DL in response to sulphur deficiency in Spark endosperm at 21 dpa was significant (*p* < 0.01 and *p* < 0.001, respectively), as was the increase in expression of the 3B homeologue in the embryo (*p* < 0.01). The increase in expression of the 4BL and 4DL homeologues of the SnRK1b* gene in response to sulphur deficiency at 21 dpa was significant in SR3 embryo (*p* = 0.0426 and *p* = 0.0377, respectively), as was the increase in expression of the 4DL homeologue in Spark endosperm (*p* = 0.0404)

Cereals contain two types of gene encoding the catalytic subunit of SnRK1, called *SnRK1a* and *SnRK1b*, with *SnRK1a* being more similar to the dicot type and *SnRK1b* appearing to be monocot-specific and expressed at high levels in the developing endosperm [31–33]. The expression of the *SnRK1* genes in this study is shown in Fig. 8. A *SnRK1a* gene was identified on chromosome 1 (1AL, 1B and 1DL) and, as shown previously, was more highly expressed in the embryo than the endosperm (Fig. 8). A second *SnRK1a* gene was identified on chromosome 3 (3AL, 3BL and 3DL) but was expressed at much lower levels than the chromosome 1 gene, with both the 3BL and 3DL homeologues encoding proteins truncated by 150 amino acids at the C-terminal end. A *SnRK1b* gene was also located on chromosome 3, but only 3B and 3DL, with no homeologue present on chromosome 3A. As expected, this gene showed much higher levels of expression in the endosperm than the embryo (Fig. 8). A second, truncated b-type gene was identified on chromosome 3B but was not expressed. A third type of *SnRK1* gene was identified on chromosome 4, with two copies on chromosome 4A and one on each of 4BL and 4DL. Of these, one of the 4A homeologues and the 4D homeologue encoded full-length proteins and were expressed in both embryo and endosperm (Fig. 8). The 4B homeologue and the other 4A homeologue were found to encode truncated proteins. Nevertheless, the 4B homeologue was expressed in similar fashion to the full-length 4A and 4D homeologues (Fig. 8). This gene type encoded a protein more similar to the *SnRK1b* type than the *SnRK1a* type, but since it showed a different expression pattern to the *SnRK1b* gene (Fig. 8) it was called SnRK1b*.

All three homeologues of the *SnRK1a* gene on chromosome 1 were more highly expressed in the embryo of Spark than SR3, particularly at 14 dpa, consistent with *TaASN2* expression. There was no consistent response to sulphur at 14 dpa, but at 21 dpa there was a trend for the *SnRK1b* and SnRK1b* genes to increase in expression in response to sulphur deprivation (Fig. 8c and d). This was significant for the *SnRK1b* homeologues on chromosomes 3B and 3DL in Spark endosperm (*p* < 0.01 and *p* < 0.001, respectively). The 3B homeologue likewise showed a low level of expression in the embryo and this also rose significantly in Spark in response to sulphur deficiency (*p* < 0.01). Expression of the 4BL and 4DL homeologues of the SnRK1b* gene rose significantly in response to sulphur deficiency in SR3 embryo (*p* = 0.0426 and 0.0377, respectively), while expression of the 4DL homeologue also rose significantly in the endosperm of Spark (*p* = 0.0404). These data added to the evidence for a much stronger response to sulphur at the later timepoint and demonstrated a clear responsiveness of *SnRK1* gene expression to sulphur, implicating this important metabolic regulator in the sulphur response.

### General control nonderepressible-2-type protein kinase (GCN2)

Another protein kinase that has been shown to play a role in regulating asparagine synthetase gene expression in wheat is GCN2, which phosphorylates translation initiation factor eIF2α. GCN2 is encoded by a single gene in every plant species in which it has been identified and is the only plant eIF2α kinase [34]. Its over-expression in transgenic wheat results in reduced total free amino acid and free asparagine concentration in the grain [35]. *TaASN1* expression in the leaves of over-expressing plants is greatly reduced and does not increase in response to sulphur deficiency, whereas it does in wild-type wheat leaves [35].

**Fig. 9:**
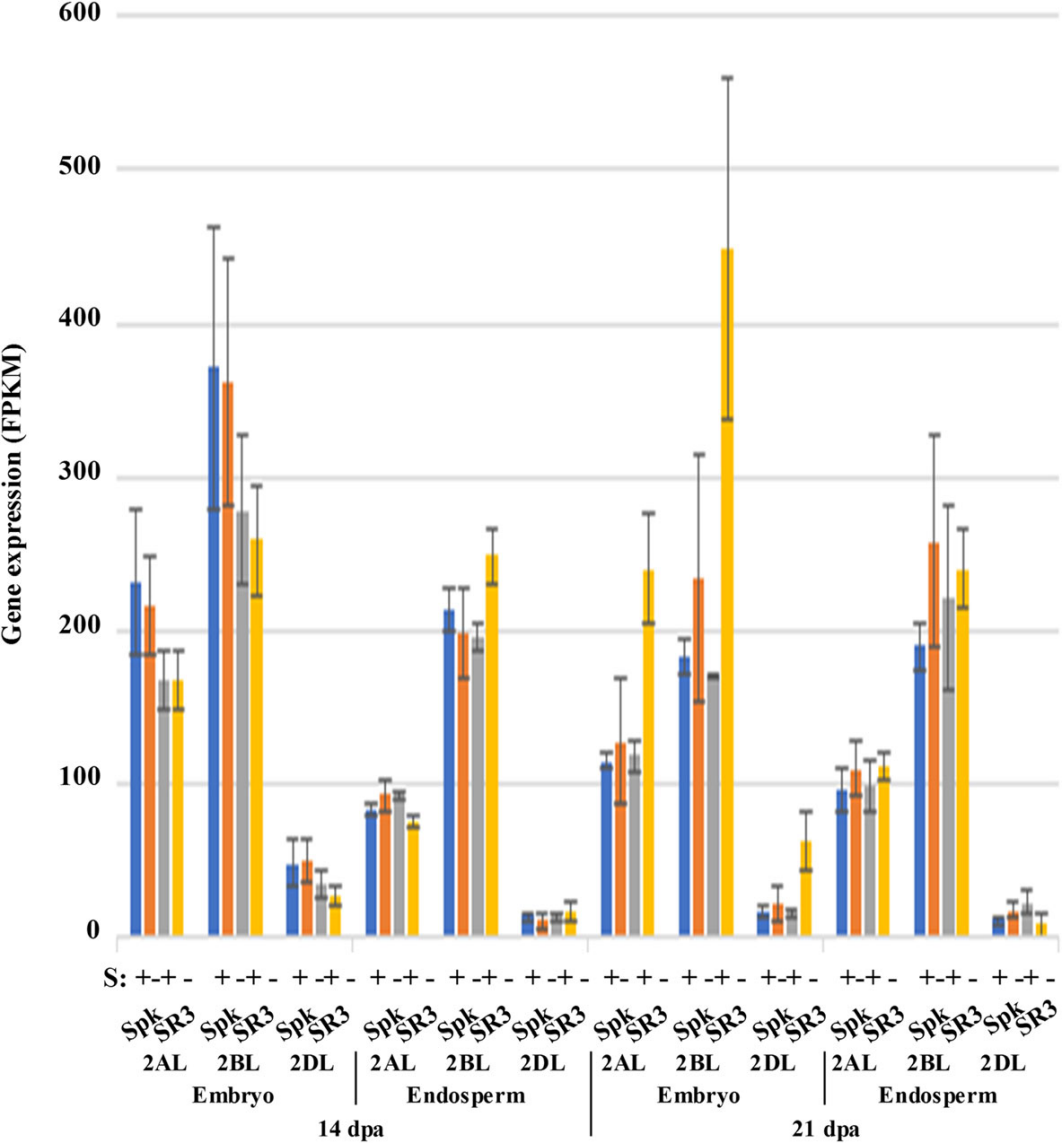
Expression levels (FPKM) of genes encoding general control nonderepressible-2 (GCN2) protein kinase in the embryo and endosperm of developing grain from wheat (*Triticum aestivum*) genotypes Spark and SR3. Plants were grown with sulphur either supplied (+) or withheld (−) and analysed at 14 and 21 dpa. Results for each homeologue are shown separately, as indicated. Gene reference numbers are given in Additional file 2. The increase in expression of all three homeologues in response to sulphur deficiency in SR3 embryos at 21 dpa was significant (*p* = 0.03114 for the 2AL homeologue, < 0.01 for the 2BL homeologue and 0.01313 for the 2DL homeologue)

Analysis of the dataset produced in this study confirmed that a single *TaGCN2* gene was present on chromosome 2 (2AL, 2BL and 2DL), with the 2BL homeologue showing the highest expression level and the 2DL homeologue showing very low levels of expression (Fig. 9). Expression of all three homeologues increased in SR3 embryos at 21 dpa under sulphur deficiency (*p* = 0.03114 for the 2AL homeologue, < 0.01 for the 2BL homeologue and 0.01313 for the 2DL homeologue). This can further explain the increased responsiveness of SR3 to sulphur.

### bZIP transcription factors

A total of 576 transcription factors (inclusive of homeologues) showed changes in gene expression in response to sulphur deficiency (Additional file 2: Table S1). Basic Leucine Zipper Domain (bZIP) transcription factors were of interest because they are involved in regulating asparagine synthetase gene expression. For example, bZIPs regulate Arabidopsis *AtASN1* gene expression via a bZIP binding site adjacent to the TATA box [30]. bZIPs are known to bind more than one target site, typically but not exclusively palindromic sequences with an ACGT core [36], and the binding site in *AtASN1* is a G-box (CACGTG). Transcription factors shown to bind this site in *AtASN1* include bZIP11, which is involved in sucrose signalling and is regulated at the translational level [37]. However, the G-box of *AtASN1* is not present in wheat asparagine synthetase gene promoters (Additional file 2: Figure S3a-c). The cDNA data that are provided for the *TaASN2* genes in the EnsemblPlants database suggest that the *TaASN2* mRNA has a long leader sequence, and the relatively short stretch of nucleotide sequence data upstream of the ATG translation start site available for the *TaASN2* gene on chromosome 3AS from the database do not include an obvious TATA box. However, the longer nucleotide sequence data for the 3DS gene do contain a putative TATA sequence 625 base pairs upstream of the ATG translation start site (Additional file 2: Figure S3d). Adjacent to this putative TATA box is a bZIP binding site, but it is a C-box (GACGTC) rather than a G-box.

The *TaASN1* gene contains a putative TATA box much closer to the ATG translation start site (Additional file 2: Figure S3a-c), but this does not have an adjacent bZIP binding site. However, a potential regulatory motif has previously been identified in *TaASN1* genes [22] and this motif, ATGAGTCATC, is present in all three *TaASN1* genes in the EnsemblPlants database (Additional file 2: Figure S3a-c). This motif is also present in cereal storage protein gene promoters, where it comprises the N-motif and is responsible for positive and negative effects on gene expression in response to nitrogen availability [38]. It is identical to the binding site for GCN4 of budding yeast, a transcription factor that is regulated at the translational level as a result of GCN2 phosphorylation of translation initiation factor eIF2α (see [34] for review). Paradoxically, plants do not have a direct homologue of GCN4, but the motif is recognised by several bZIP transcription factors, including Opaque2 of maize [39], Opaque2 dimerising protein (OHP1/BLZ1) [40, 41] and SPA [42].

**Fig. 10:**
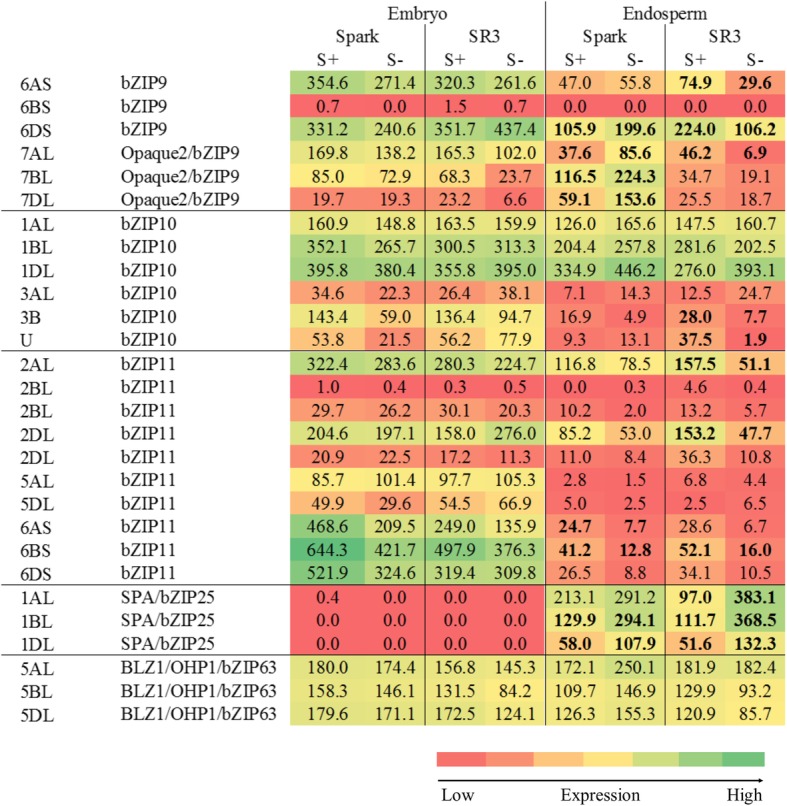
Heatmap representing relative expression levels (FPKM) of genes encoding bZIP transcription factors bZIP9, Opaque2/bZIP9, bZIP10, bZIP11, SPA/bZIP25 and BLZ1/OHP1/bZIP63 classes in the embryo and endosperm of developing grain from wheat (*Triticum aestivum*) genotypes Spark and SR3 at 21 dpa. Plants were grown with sulphur either supplied (S+) or withheld (S-). Results for each homeologue are shown separately, as indicated. Gene reference numbers are given in Additional file 2. Significant (*p* < 0.05) changes in expression in response to sulphur are shown in bold

Genes encoding these transcription factors were identified in the dataset and a heatmap of their expression at 21 dpa, when some sulphur responses became evident, is shown in Fig. 10. A gene encoding an Opaque2-like transcription factor was identified on chromosome 7 (7AL, 7BL and 7DL), although it was annotated in the EnsemblPlants database simply as *bZIP9*. A gene on chromosome 6 (6AS, 6BS and 6DS) was also annotated as *bZIP9*, and although it encodes a transcription factor that is less similar to Opaque2, it was also included in the heatmap. A gene encoding the SPA transcription factor was identified on chromosome 1 (1AL, 1BL and 1DL), annotated as *bZIP25*, while a gene encoding an OHP1/BLZ1-like transcription factor was identified on chromosome 5 (5AL, 5BL and 5DL), annotated as *bZIP63*. Notably, bZIP9, bZIP25 and bZIP63 are implicated in the regulation of asparagine synthetase gene expression in Arabidopsis, along with bZIP11 and bZIP10 [30, 37], although their similarity to Opaque2, SPA and OHP1/BLZ1 has not been discussed previously to our knowledge. Data on expression of *bZIP10* genes (chromosomes 1 and 3) and *bZIP11* genes (chromosomes 2, 5 and 6) are included in the heat map.

These transcription factors all have potential sites for phosphorylation by SnRK1, with the exception of bZIP11, which is translationally regulated [37]. SnRK1 preferentially phosphorylates a serine residue with a hydrophobic residue at + 4 and − 5 with respect to the serine and a basic residue at − 3, or less preferably at − 4 [43]. The Opaque2/bZIP9 protein has such a sequence (**Met**-Lys-**Lys**-Cys-Ala-**Ser**-Glu-Leu-Glu-**Leu**) at its N-terminal end, with the target serine at position 6, while bZIP10 has the sequence **Leu**-Ala-**Arg**-Phe-Arg-**Ser**-Ala-Ser-Gly-**Ile** with the target serine at position 26. The SPA/bZIP25 protein has the site **Leu**-**Arg**-Ile-Pro-Phe-**Ser**-Gly-Ser-Pro-**Leu** with the target serine at position 233, while the OHP1/BLZ1/bZIP63 protein has two target sites in its N-terminus (positions 6 and 39) and two overlapping sites in its C-terminal region (position 367 and 373) [44].

The heatmap (Fig. 10) shows differential expression between the homeologues and the genotypes for the *Opaque2/bZIP9* gene on chromosome 7, with the 7A gene expressed more highly in the embryo than the endosperm but the other two being expressed more highly in the endosperm than the embryo in Spark but at similar levels in both tissues in SR3. Expression of all three homeologues increased significantly in response to sulphur deprivation in the endosperm of Spark (*p* < 0.01 for the 7AL homeologue; *p* = 0.0398 and 0.0405, respectively, for the 7BL and 7DL homeologues), but the 7A gene decreased in expression in SR3 endosperm in response to sulphur deficiency (*p* < 0.001) while the other two homeologues showed no significant change (*p* > 0.05). The 6A and 6D homeologues of the other *bZIP9* gene showed similar expression patterns, while the 6B homeologue was hardly expressed at all.

The *bZIP10* gene on chromosome 3 was expressed at low levels in both tissues, but the 3B homeologue and an unassigned gene that was almost certainly the 3D homeologue did show a significant reduction in expression (*p* = 0.367 and *p* < 0.001, respectively, for the 3B and 3D homeologues), albeit from already low levels, in response to sulphur deficiency in SR3. The *bZIP10* gene on chromosome 1, on the other hand, was expressed at relatively high levels in both tissues but showed no response to sulphur.

The *bZIP11* genes on chromosome 2 showed differential expression between homeologues, with both genes on chromosome 2B and one of the genes on 2D expressed at low levels, but the other 2D gene and the only gene on chromosome 2A expressed at relatively high levels particularly in the embryo. These two genes also showed reduced expression in the endosperm in response to sulphur deficiency, but this was significant only in SR3 (*p* < 0.001 for the 2AL and 2DL homeologues). Neither homeologue of the *bZIP11* gene on chromosome 5 was highly expressed, but both (5AL and 5DL) were expressed at higher levels in the embryo than the endosperm. The most highly expressed of the *bZIP11* genes was the one on chromosome 6 (6AS, 6BS and 6DS). As with the other *bZIP11* genes, there was higher expression in the embryo than the endosperm, and there was also a trend for lower expression in the endosperm in response to sulphur deficiency. This was significant (*p* = 0.0419) for the 6AS homeologue in Spark and for the 6BS homeologue in both genotypes (*p* = 0.0229 for Spark and 0.03903 for SR3).

The *SPA/bZIP25* gene on chromosome 1 was expressed in the endosperm but not at all in the embryo. Expression of all three homeologues increased significantly at 21 dpa in SR3 endosperm in response to sulphur deficiency (*p* < 0.01), while the 1BL and 1DL homeologues also increased in Spark (*p* = 0.03 for both). The endosperm-specific expression of this transcription factor would be consistent with a role in regulating storage protein gene expression but suggests that it is not involved in regulating asparagine synthetase gene expression.

The *OHP1/BLZ1/bZIP63* transcription factor gene on chromosome 5, on the other hand, was expressed at similar levels in both endosperm and embryo, and clearly its expression in the embryo suggests that regulating storage protein gene expression is not its only role. It showed no response to sulphur, but as with all of the transcription factors its primary regulation may be post-transcriptional; indeed, this seems likely given its multiple SnRK1 target sites.

## Discussion

The first observation to be made on the data is that there were clear differences between the genotypes with respect to the expression of key genes involved in asparagine metabolism. Secondly, sulphur responses were much more evident at 21 dpa than 14 dpa, and much more evident in SR3 than Spark. Both genotypes have been shown previously to respond to sulphur deficiency with a massive accumulation of free asparagine in the grain [6], so this was unexpected. It is possible that Spark was simply behind SR3 developmentally and 21 dpa was too early to see the S response in this genotype.

The study confirmed *TaASN2* to be the most highly expressed asparagine synthetase gene in the grain, with expression in the embryo much higher than in the endosperm. Indeed, the data suggest strongly that the embryo is the organ in which grain asparagine levels are determined. An obvious possible explanation of why Spark accumulates more free asparagine in the grain than SR3 was provided by the higher expression of *TaASN2* in Spark during early development (14 dpa), and there was also a trend for genes encoding enzymes of nitrogen assimilation to be more highly expressed in Spark than SR3 when sulphur was supplied, consistent with the higher levels of total free amino acids in the grain of that genotype. Interestingly, genes encoding both nitrate reductase and nitrite reductase were expressed, suggesting that nitrate must be imported into the grain.

*TaASN2* and glutamine synthetase gene expression in the embryo of SR3 increased in response to sulphur deficiency at 21 dpa, while asparaginase gene expression decreased. Asparagine synthetase and asparaginase gene expression in the endosperm responded in the opposite way, falling and rising, respectively, in response to sulphur deficiency. This suggests that the asparagine that accumulates in the endosperm in response to sulphur deficiency is imported from the embryo or elsewhere. We speculate that asparaginase is expressed in readiness to remobilise the free asparagine if sulphur becomes available, for example if the roots reach a source of sulphur in the soil, or at germination. This would mean that the enzyme would have to be inactive until required.

Another notable observation was the expression of regulatory protein kinases, SnRK1 and GCN2. Both have been implicated in regulating asparagine synthetase gene expression before [30, 35], and both showed responses to sulphur deficiency that would be consistent with that role. bZIP transcription factors, including four with SnRK1 target sites, were also expressed, and putative regulatory motifs at which these transcription factors could bind were identified in both *TaASN1* and *TaASN2* promoters, including the N-motif identified previously in *TaASN1* [22]. Three of these transcription factors, Opaque2/bZIP9, SPA/bZIP25 and BLZ1/OHP1/bZIP63, are known to bind the N-motif. The data are certainly consistent with SnRK1 regulating *TaASN1* and *TaASN2* expression through these transcription factors.

Another general observation from the data was that the homeologues of many genes showed differential expression patterns and responses. In the context of the aims of the study, this was most important for *TaASN2*, the chromosome 3A homeologue of which was expressed at much higher levels than the 3D homeologue, while the 3B homeologue was either not expressed or was missing altogether.

## Conclusions

The study provided extensive new data on the genetic control of free asparagine accumulation in wheat grain and its response to sulphur supply. It showed the embryo to be the organ in which grain asparagine levels are determined, based on the levels of expression of the key genes involved, notably those encoding asparagine synthetase, and identified genes encoding signalling and metabolic proteins involved in asparagine metabolism that respond to sulphur availability. Asparagine synthetase gene *TaASN2* was confirmed as a logical target for genetic interventions aimed at reducing the asparagine content of wheat and other cereal grains, and the data suggested that interventions aimed at the A genome homeologue alone could be effective. The study also identified genes encoding other metabolic enzymes and signalling factors that could be targeted.

## Methods

### Sample preparation

Wheat plants of variety Spark and doubled haploid line SR3 from a Spark × Rialto mapping population [16] were grown from seed held at Rothamsted Research by Tanya Curtis and Nigel Halford. The seed were derived from lines originally supplied by the John Innes Centre Wheat Genetics Group, Norwich, UK. The plants were grown in vermiculite in a glasshouse with a 16 h day-length (supplemental lighting was used as necessary) and a minimum temperature of 16 °C. Vermiculite does not retain nutrients, so the only nutrition available to the plants came from liquid feed solution. Feeding was started 3 weeks after potting and continued every 2 days until harvest. Plants were supplied with either a medium containing a full nutrient complement of potassium, phosphate, calcium, magnesium, sodium, iron, nitrate (2 mM Ca(NO_3_)_2_ and 1.6 mM Mg(NO_3_)_2_) and sulphate ions (1.1 mM MgSO_4_) [4, 6], or the same medium containing one tenth the concentration of MgSO_4_. Distilled water was supplied as required to prevent water stress. A randomised design was used for the pots in the glasshouse.

Ears were tagged at anthesis, grain sampled at 14 and 21 dpa, and caryopses dissected under a microscope to isolate the embryo and endosperm. Dissected samples were immediately frozen in liquid nitrogen and stored at − 80 °C. There were four biological replicates for each of the two time-points, two treatments and two varieties, making a total of 32 embryo and 32 endosperm samples.

### RNA extraction and RNA-seq analysis

The RNA extraction method was modified from Chang et al. [45]. Frozen tissues were ground in liquid nitrogen and extracted in CTAB buffer (2% (w/v) cetyl trimethylammonium bromide, 2% (w/v) polyvinylpyrrolidone (PVP) K 30, 100 mM Tris-HCl, pH 8.0, 25 mM ethylenediaminetetraacetic acid (EDTA), 2.0 M NaCI, 0.5 g/L spermidine, 2% (w/v) β-mercaptoethanol). The supernatant was extracted twice with chloroform: isoamyl alcohol (IAA) (24:1) to remove proteins. RNA was precipitated by addition of 0.25 vol. of 10 M LiCl and incubation on ice overnight. The RNA pellet was dissolved in SSTE buffer (1.0 M NaCl, 0.5% (w/v) SDS, 10 mM Tris HCl pH 8.0, 1 mM EDTA) to remove polysaccharides and extracted once with chloroform: isoamyl alcohol. After ethanol precipitation, total RNA was dissolved in diethyl pyrocarbonate-treated water and stored at − 80 °C. Total RNA was treated with deoxyribonuclease and purified through RNeasy mini spin columns (Qiagen, Crawley, UK).

The RNA-seq analysis was performed by GATC Biotech (Konstanz, Germany) using the NGSelect option, generating 15 million 125 bp paired-end strand-specific reads per sample.

### Bioinformatics

The reads were not trimmed. HISAT2 (v2.0.5) was used to map the RNA-seq data to the most comprehensive available *Triticum aestivum* cv. Chinese Spring reference at the time of analysis (TGACv1) with FeatureCounts (v1.5.1) used to count against the reference annotation exons using the strand-specific option. Mapping rates across samples for SR3 were: 1,364,906,937 in total; 1,225,035,426 properly paired (89.75%); 50,200,825 singletons (3.68%); 22,083,695 with mate mapped to a different chromosome (mapQ> = 5). For Spark the mapping rates were: 1,514,802,531 in total; 1,349,695,534 properly paired (89.10%); 62,138,374 singletons (4.10%); 29,187,503 with mate mapped to a different chromosome (mapQ> = 5). The R package DeSeq2 (v1.22.2) was used for differential expression and genes functionally annotated using Blast2Go (v4.2). Genes with an adjusted *p*-value of < 0.05 were regarded as differentially expressed.

Gene Ontology enrichment analysis was performed using the Fisher’s Exact Test in Blast2GO version 5.2.5 [46]. The enrichment analysis was limited to Molecular Function and Biological Process categories and the FDR threshold was set to 0.05. The results were summarised further to the most specific GO term.

## Additional files

Additional file 1: Results of functional enrichment analysis. (XLSX 336 kb)

Additional file 2: EnsemblPlants reference numbers for genes discussed in the paper. **Figure S1.** Expression levels (FPKM) of genes encoding aspartate kinase. **Figure S2.** Expression levels (FPKM) of genes encoding glutamate synthase (GOGAT). **Figure S3.** Asparagine synthetase gene promoter nucleotide sequences. **Table S1.** Transcription factors differentially expressed in response to sulphur deficiency. (DOCX 1653 kb)

## Data Availability

The raw data has been deposited in European Nucleotide Archive (ENA) and is publicly available (https://www.ebi.ac.uk/ena) using the study accession: PRJEB31122.

## Abbreviations

bZIP: Basic leucine zipper
EFSA: European Food Safety Authority
FPKM: Fragments per kilobase of transcript per million mapped reads
GCN2: General control nonderepressible-2
GOGAT: Glutamate synthase (glutamine 2-oxyoglutarate aminotransferase)
RNA-seq: RNA sequencing
SNF1: Sucrose nonfermenting-1
SnRK1: SNF1-related protein kinase-1
