# Supplementary material for: Contrasting gene expression patterns in grain of high and low asparagine wheat genotypes in response to sulphur supply
Source: BMC Genomics. 2019 Aug 1;20:628. doi: 10.1186/s12864-019-5991-8 (PMC6676566; doi:10.1186/s12864-019-5991-8)
Supplement: Supplementary file 2 — EnsemblPlants reference numbers for genes discussed in the paper. Figure S1. Expression levels (FPKM) of genes encoding aspartate kinase. Figure S2. Expression levels (FPKM) of genes encoding glutamate synthase (GOGAT). Figure S3. Asparagine synthetase gene promoter nucleotide sequences. Table S1. Transcription factors differentially expressed in response to sulphur deficiency. (DOCX 1653 kb) [file 12864_2019_5991_MOESM2_ESM.docx]

**Contrasting gene expression patterns in grain of high and low asparagine wheat genotypes in response to sulphur supply**

**Tanya Y. Curtis ^1^, Sarah Raffan^1^, Yongfang Wan^1^, Robert King^2^, Asier Gonzalez-Uriarte^2^ and Nigel G. Halford^1*^**

***^1^Plant Sciences Department, Rothamsted Research, Harpenden, Hertfordshire AL5 2JQ, United Kingdom***

***^2^Computational and Analytical Sciences Department, Rothamsted Research, Harpenden, Hertfordshire AL5 2JQ, United Kingdom***

^*^To whom correspondence should be addressed: Telephone: 44 1582 938203. Email: [nigel.halford@rothamsted.ac.uk](mailto:nigel.halford@bbsrc.ac.uk)

**ADDITIONAL FILE 2**

**EnsemblPlants reference numbers**

Reference numbers for asparagine synthetase genes

*TaASN1*

TRIAE_CS42_5AL_TGACv1_376022_AA1230940

TRIAE_CS42_5BL_TGACv1_404794_AA1310950 TRIAE_CS42_5DL_TGACv1_438333_AA1468150

*TaASN2*

TRIAE_CS42_3AS_TGACv1_210989_AA0682630 TRIAE_CS42_3DS_TGACv1_271746_AA0907290

*TaASN3.1*

TRIAE_CS42_1AL_TGACv1_004377_AA0053370

TRIAE_CS42_1BL_TGACv1_032370_AA0129050 TRIAE_CS42_1DL_TGACv1_061978_AA0206740

*TaASN3.2*

TRIAE_CS42_1AL_TGACv1_002273_AA0040340 TRIAE_CS42_1BL_TGACv1_031075_AA0107150 TRIAE_CS42_1DL_TGACv1_061247_AA0189910

*TaASN4*

TRIAE_CS42_4AS_TGACv1_308427_AA1027880 TRIAE_CS42_U_TGACv1_641929_AA2107490 TRIAE_CS42_4DL_TGACv1_342578_AA1117080

Reference numbers for asparaginase genes:

TRIAE_CS42_2AL_ TGACv1_093489_AA0281040

TRIAE_CS42_2AL_ TGACv1_094214_AA0294520

TRIAE_CS42_2AL_ TGACv1_094425_AA0297410

TRIAE_CS42_2AL_ TGACv1_095089_AA0306740

TRIAE_CS42_2AL_ TGACv1_095635_AA0313170

TRIAE_CS42_2AL_ TGACv1_096004_AA0316590

TRIAE_CS42_2BL_ TGACv1_129295_AA0377030

TRIAE_CS42_2BL_ TGACv1_129317_AA0378360

TRIAE_CS42_2BL_ TGACv1_130210_AA0406380

TRIAE_CS42_2BL_ TGACv1_131069_AA0421990

TRIAE_CS42_2BL_ TGACv1_131110_AA0422710

TRIAE_CS42_2DL_ TGACv1_159434_AA0538280

TRIAE_CS42_2DL_ TGACv1_160859_AA0554720

TRIAE_CS42_2DL_ TGACv1_161315_AA0557930

TRIAE_CS42_2DL_ TGACv1_161808_AA0560690

TRIAE_CS42_2DL_ TGACv1_158126_AA0509920

TRIAE_CS42_U_ TGACv1_683995_AA215903

TRIAE_CS42_U_ TGACv1_686553_AA2159690)

TRIAE_CS42_3AS_ TGACv1_211351_AA0688980

TRIAE_CS42_3B_ TGACv1_223674_AA0785450

TRIAE_CS42_3DS_ TGACv1_272245_AA0917710

Reference numbers for aspartate kinase (ASPK) genes:

TRIAE_CS42_3AL_TGACv1_193656_AA0616610

TRIAE_CS42_3B_TGACv1_224879_AA0802660

TRIAE_CS42_3DL_TGACv1_251860_AA0885520

TRIAE_CS42_4AL_TGACv1_290400_AA0985020

TRIAE_CS42_5BL_TGACv1_404409_AA1298740

TRIAE_CS42_5DL_TGACv1_433910_AA1425110

TRIAE_CS42_5DL_TGACv1_439458_AA1468950

Reference numbers for aspartate aminotransferase genes:

TRIAE_CS42_3AS_TGACv1_211341_AA0688770

TRIAE_CS42_3B_TGACv1_222042_AA0755620

TRIAE_CS42_3DS_TGACv1_272451_AA0920870

Reference numbers for bZIP genes:

*bZIP9*

TRIAE_CS42_6AS_TGACv1_488428_AA1575500

TRIAE_CS42_6BS_TGACv1_515508_AA1670570

TRIAE_CS42_6DS_TGACv1_545295_AA1750190

TRIAE_CS42_7AL_TGACv1_556285_AA1759970

TRIAE_CS42_7BL_TGACv1_577418_AA1875080

TRIAE_CS42_7DL_TGACv1_603981_AA1991930

*bZIP10*

TRIAE_CS42_1AL_TGACv1_000812_AA0019550

TRIAE_CS42_1BL_TGACv1_031237_AA0110100

TRIAE_CS42_1DL_TGACv1_061988_AA0206970

TRIAE_CS42_3AL_TGACv1_197036_AA0664480

TRIAE_CS42_3B_TGACv1_221698_AA0747790

TRIAE_CS42_U_TGACv1_643015_AA2126130

*bZIP11*

TRIAE_CS42_2AL_TGACv1_093869_AA0288470

TRIAE_CS42_2BL_TGACv1_129307_AA0377660

TRIAE_CS42_2BL_TGACv1_132950_AA0440660

TRIAE_CS42_2DL_TGACv1_157989_AA0504940

TRIAE_CS42_2DL_TGACv1_160949_AA0555500

TRIAE_CS42_5AL_TGACv1_376957_AA1242740

TRIAE_CS42_5DL_TGACv1_433913_AA1425130

TRIAE_CS42_6AS_TGACv1_487131_AA1568440

TRIAE_CS42_6BS_TGACv1_513373_AA1639310

TRIAE_CS42_6DS_TGACv1_542930_AA1732700

*bZIP25*

TRIAE_CS42_1AL_TGACv1_000470_AA0012860

TRIAE_CS42_1BL_TGACv1_030508_AA0092770

TRIAE_CS42_1DL_TGACv1_061220_AA0189160

*bZIP63*

TRIAE_CS42_5AL_TGACv1_375799_AA1227320

TRIAE_CS42_5BL_TGACv1_408138_AA1361800

TRIAE_CS42_5DL_TGACv1_433372_AA1411290

Reference numbers for CBL-interacting protein kinase (CIPK) genes:

*CIPK2*

TRIAE_CS42_2AS_TGACv1_112595_AA0341820

TRIAE_CS42_2BS_TGACv1_147085_AA0477750

*CIPK6*

TRIAE_CS42_4AL_TGACv1_289706_AA0975620

TRIAE_CS42_4BS_TGACv1_328536_AA1089790

TRIAE_CS42_4DS_TGACv1_362508_AA1180190

*CIPK7*

TRIAE_CS42_5BL_TGACv1_404158_AA1287310

TRIAE_CS42_5DL_TGACv1_433934_AA1425570

TRIAE_CS42_5DL_TGACv1_433934_AA1425590

TRIAE_CS42_U_TGACv1_644432_AA2139480

TRIAE_CS42_U_TGACv1_644432_AA2139490

*CIPK8*

TRIAE_CS42_3DL_TGACv1_249774_AA0856020

*CIPK9*

TRIAE_CS42_4BL_TGACv1_322341_AA1070740

TRIAE_CS42_5AL_TGACv1_375277_AA1218980

TRIAE_CS42_U_TGACv1_644157_AA2137680

*CIPK11*

TRIAE_CS42_3AL_TGACv1_194451_AA0633300

TRIAE_CS42_3B_TGACv1_220872_AA0721830

TRIAE_CS42_3DL_TGACv1_249796_AA0856540

*CIPK14*

TRIAE_CS42_4AL_TGACv1_288973_AA0962350

TRIAE_CS42_4BS_TGACv1_330992_AA1109300

TRIAE_CS42_4DS_TGACv1_363325_AA1183670

TRIAE_CS42_5BL_TGACv1_409341_AA1365820

*CIPK15*

TRIAE_CS42_5AL_TGACv1_374165_AA1192190

TRIAE_CS42_5DL_TGACv1_433774_AA1421700

*CIPK16*

TRIAE_CS42_5AL_TGACv1_373966_AA1185640

TRIAE_CS42_5BL_TGACv1_405924_AA1337810

TRIAE_CS42_5DL_TGACv1_434002_AA1427040

*CIPK17*

TRIAE_CS42_1AS_TGACv1_019197_AA0062840

TRIAE_CS42_1BS_TGACv1_049662_AA0159060

TRIAE_CS42_1DS_TGACv1_081258_AA0259330

*CIPK19*

TRIAE_CS42_1AL_TGACv1_002358_AA0041150

TRIAE_CS42_1BL_TGACv1_030463_AA0091500

TRIAE_CS42_1DL_TGACv1_061299_AA0191750

TRIAE_CS42_3AL_TGACv1_199052_AA0669360

TRIAE_CS42_3B_TGACv1_223231_AA0778550

TRIAE_CS42_U_TGACv1_641330_AA2092140

*CIPK21*

TRIAE_CS42_2AS_TGACv1_113633_AA0358690

TRIAE_CS42_2BS_TGACv1_148708_AA0494740

TRIAE_CS42_2DS_TGACv1_178116_AA0590910

*CIPK23*

TRIAE_CS42_2AL_TGACv1_094301_AA0295500

TRIAE_CS42_2BL_TGACv1_129359_AA0380140

TRIAE_CS42_2DL_TGACv1_158556_AA0521800

*CIPK24*

TRIAE_CS42_7BL_TGACv1_577082_AA1865520

TRIAE_CS42_7DL_TGACv1_602749_AA1967290

*CIPK25*

TRIAE_CS42_7AL_TGACv1_557476_AA1781770

TRIAE_CS42_7BL_TGACv1_576746_AA1853220

TRIAE_CS42_7DL_TGACv1_605213_AA2005430

*CIPK26*

TRIAE_CS42_6AS_TGACv1_488069_AA1574220

TRIAE_CS42_6BS_TGACv1_514774_AA1664220

*CIPK27*

TRIAE_CS42_5AL_TGACv1_379335_AA1256100

TRIAE_CS42_5BL_TGACv1_406736_AA1349380

TRIAE_CS42_5DL_TGACv1_432949_AA1395710

*CIPK28*

TRIAE_CS42_1AL_TGACv1_000388_AA0010900

TRIAE_CS42_1DL_TGACv1_061701_AA0201940

*CIPK29*

TRIAE_CS42_2AS_TGACv1_114828_AA0369560

TRIAE_CS42_2BS_TGACv1_146593_AA0468910

*CIPK31*

TRIAE_CS42_2AS_TGACv1_112492_AA0339210

TRIAE_CS42_2BS_TGACv1_148148_AA0490900

TRIAE_CS42_2DS_TGACv1_178522_AA0597030

TRIAE_CS42_4AS_TGACv1_306509_AA1009420

TRIAE_CS42_4BL_TGACv1_321295_AA1058570

TRIAE_CS42_4DL_TGACv1_342497_AA1115230

*CIPK32*

TRIAE_CS42_4AL_TGACv1_289299_AA0968700

TRIAE_CS42_4BS_TGACv1_327875_AA1077320

TRIAE_CS42_4DS_TGACv1_362084_AA1176570

Reference numbers for ethylene insensitive 3-like 5 gene:

TRIAE_CS42_2DS_TGACv1_180205_AA0610430

Reference numbers for general control nonderepressible 2 (GCN2) genes:

TRIAE_CS42_2AL_TGACv1_094876_AA0304340

TRIAE_CS42_2BL_TGACv1_129909_AA0399260

TRIAE_CS42_2DL_TGACv1_161701_AA0560150

Reference numbers for glutamate decarboxylase genes:

TRIAE_CS42_3AL_TGACv1_193810_AA0620090

TRIAE_CS42_3AS_TGACv1_211026_AA0683390

TRIAE_CS42_3B_TGACv1_221158_AA0732140

TRIAE_CS42_3B_TGACv1_222610_AA0767770

TRIAE_CS42_3DL_TGACv1_249343_AA0845910

TRIAE_CS42_3DS_TGACv1_272233_AA0917470

Not shown in the paper

TRIAE_CS42_4AL_TGACv1_291775_AA0996540

TRIAE_CS42_4BL_TGACv1_320276_AA1033600

TRIAE_CS42_4BS_TGACv1_331478_AA1110020

TRIAE_CS42_4DS_TGACv1_363000_AA1182990

Reference numbers for glutamate dehydrogenase genes:

*GDH2*

TRIAE_CS42_2AL_TGACv1_094298_AA0295460

TRIAE_CS42_2BL_TGACv1_129728_AA0393950

TRIAE_CS42_2DL_TGACv1_159307_AA0536210

*GDH1*, not shown in the paper

TRIAE_CS42_3DL_TGACv1_252016_AA0887090

TRIAE_CS42_5AL_TGACv1_374507_AA1201910

TRIAE_CS42_5AL_TGACv1_374813_AA1209450

TRIAE_CS42_5BL_TGACv1_406581_AA1347380 TRIAE_CS42_5DL_TGACv1_435748_AA1454370

TRIAE_CS42_6BL_TGACv1_500257_AA1602300

TRIAE_CS42_7BS_TGACv1_592638_AA1942060

Reference numbers for glutamate synthase (NADH-dependent) genes:

TRIAE_CS42_3AL_TGACv1_193640_AA0616020

TRIAE_CS42_3B_TGACv1_220588_AA0709800

TRIAE_CS42_U_TGACv1_654540_AA2150720

Reference numbers for glutamine synthetase genes:

*GS1*

TRIAE_CS42_1AL_TGACv1_000189_AA0005730

TRIAE_CS42_1BL_TGACv1_031508_AA0115210

*GS1c*

TRIAE_CS42_4AL_TGACv1_288277_AA0943450

TRIAE_CS42_4BS_TGACv1_328907_AA1095330

TRIAE_CS42_4DS_TGACv1_361179_AA1162880

TRIAE_CS42_6AL_TGACv1_470941_AA1499070

TRIAE_CS42_6BL_TGACv1_500652_AA1608010

TRIAE_CS42_6DL_TGACv1_527226_AA1700820

*GSr1*

TRIAE_CS42_4AS_TGACv1_307728_AA1023060

TRIAE_CS42_4BL_TGACv1_320919_AA1051690

TRIAE_CS42_4DL_TGACv1_343321_AA1133210

*GS2p*

TRIAE_CS42_U_TGACv1_640900_AA2078630

TRIAE_CS42_2BL_TGACv1_129656_AA0391860

TRIAE_CS42_2DL_TGACv1_161369_AA0558280

Reference numbers for late embryogenesis abundant 12:

TRIAE_CS42_5BL_TGACv1_404414_AA1298930

Reference numbers for the low molecular weight glutenin subunit gene:

TRIAE_CS42_1BS_TGACv1_052225_AA0181540

Reference numbers for nitrate reductase genes:

[NAD(P)H]-dependent

TRIAE_CS42_6AL_TGACv1_472117_AA1517910

TRIAE_CS42_6BL_TGACv1_500036_AA1597670

TRIAE_CS42_6DL_TGACv1_528127_AA1711800

[NADH]-dependent

TRIAE_CS42_6AS_TGACv1_486190_AA1557980

TRIAE_CS42_6BS_TGACv1_514925_AA1665930

TRIAE_CS42_6DS_TGACv1_543770_AA1744010

Reference numbers for nitrite reductase genes:

TRIAE_CS42_1AL_TGACv1_001652_AA0033600

TRIAE_CS42_1BL_TGACv1_031850_AA0121710

TRIAE_CS42_1DL_TGACv1_061476_AA0196370

TRIAE_CS42_6AL_TGACv1_471208_AA1504680

TRIAE_CS42_6BL_TGACv1_499968_AA1596170

TRIAE_CS42_6DL_TGACv1_526318_AA1679410

**Figure S1**

Expression levels (fragments per kilobase of transcript per million mapped reads; FPKM) of genes encoding aspartate kinase in the embryo and endosperm of developing grain from wheat (*Triticum aestivum*) genotypes Spark and SR3. Plants were grown with sulphur either supplied (+) or withheld (-) and analysed at 14 dpa (upper panel) and 21 dpa (lower panel). Results for each homeologue are shown separately, as indicated. Gene reference numbers are given above.

**
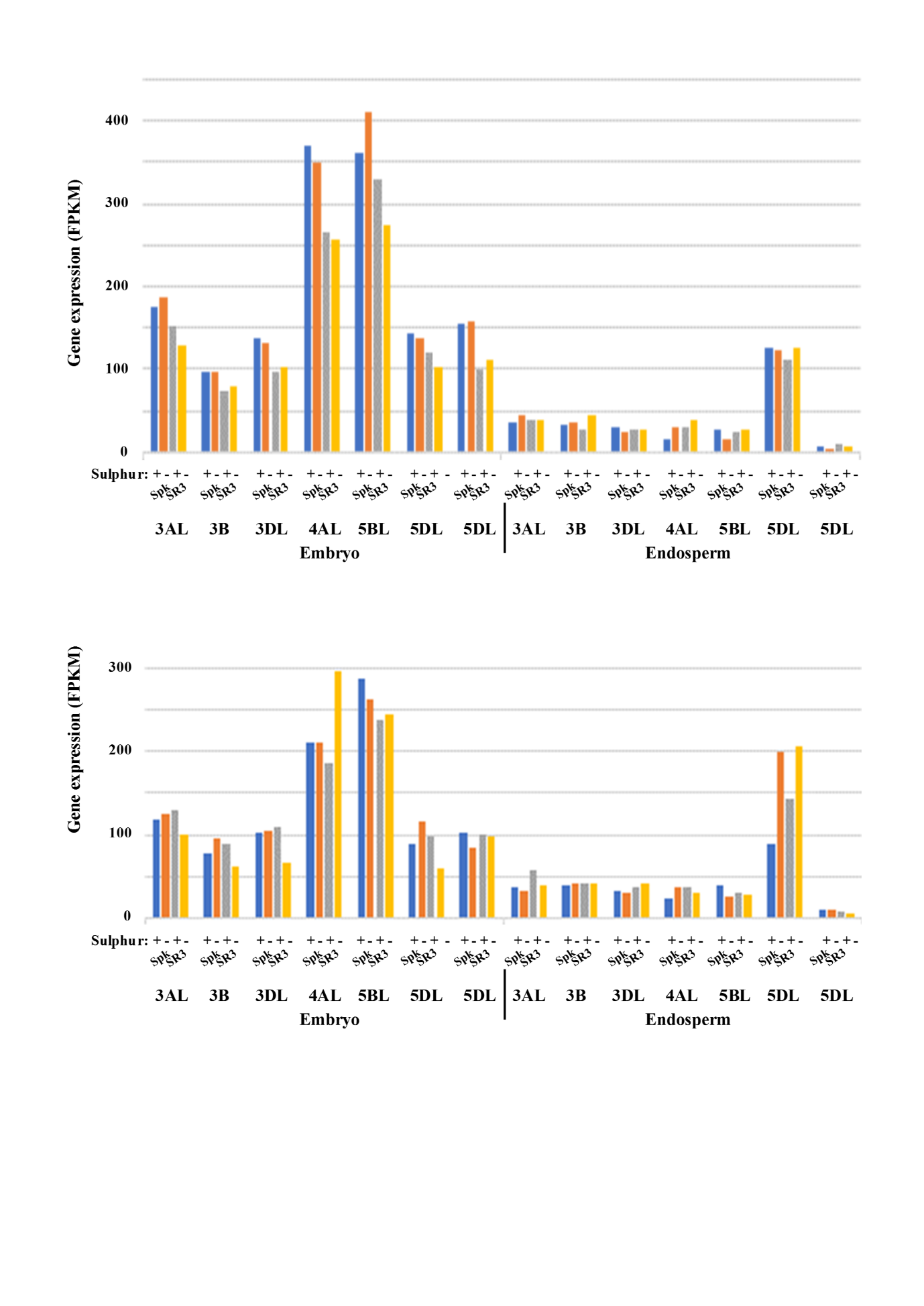
**

**Figure S2**

Expression levels (FPKM) of genes encoding glutamate synthase (GOGAT) in the embryo and endosperm of developing grain from wheat (*Triticum aestivum*) genotypes Spark and SR3. Plants were grown with sulphur either supplied (+) or withheld (-) and analysed at 14 and 21 dpa. Results for each homeologue are shown separately, as indicated. Gene reference numbers are given above.


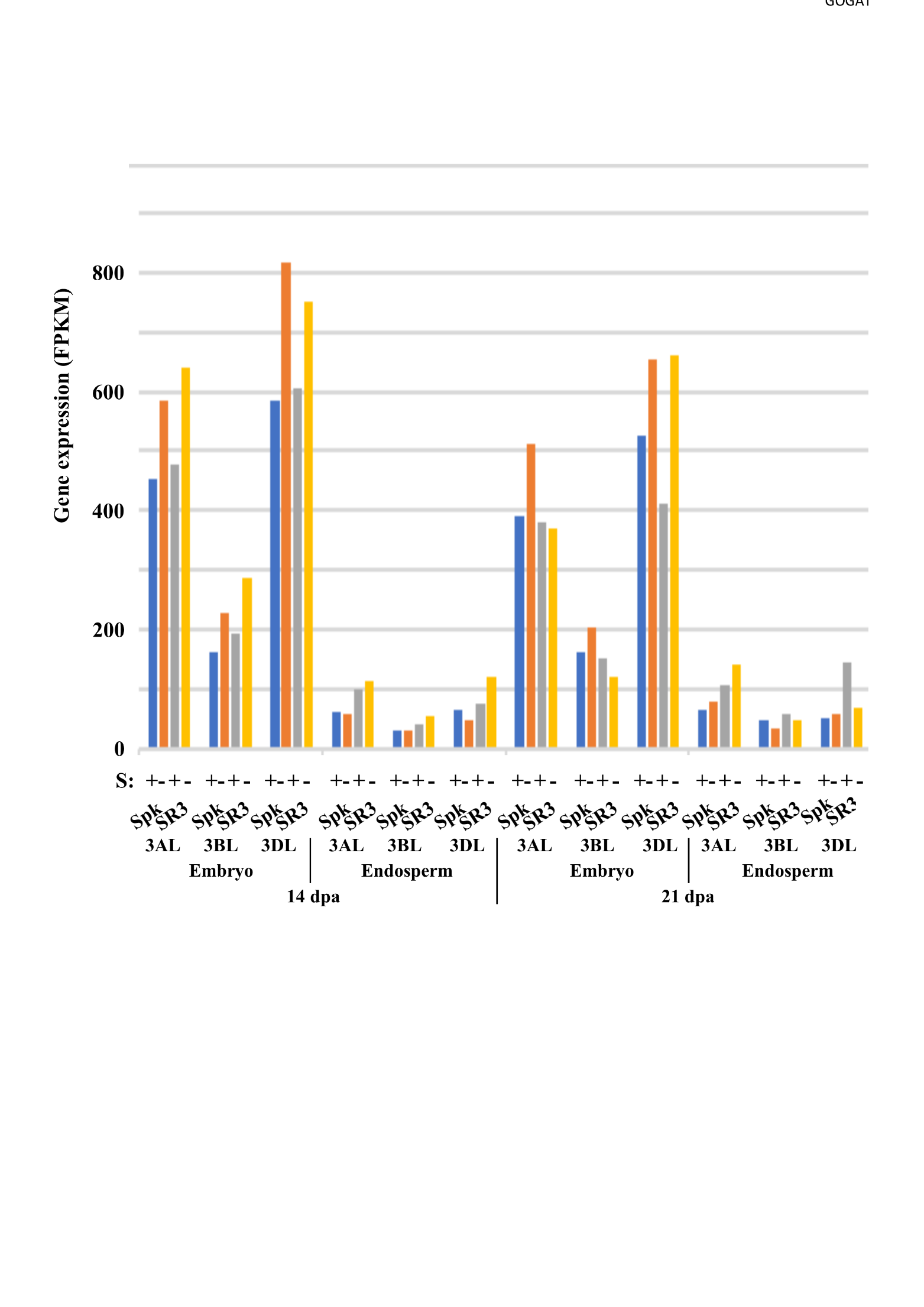


**Figure S3**

**Asparagine synthetase gene promoter nucleotide sequences**

**a. *TaASN1* gene promoter from TRIAE_CS42_5AL_TGACv1_376022_AA1230940. The ATG translation start site is highlighted in yellow, the putative TATA box is underlined and the putative N-motif is highlighted in red.**

AGCATAGCATATTCATTCAAGGAGATCCGGATCACCCAACGAGATGCACCCCTTACATAC

AGATAACAAGTACTCCCTCCGTCCGAAAATAGTTGTCATCAAAAAGAATAAAAGGGAATG

TATCTAGATGTATTTTAGTTCTAGATACATCTCTTTTCATCCATTTTGATGACAACTATT

TTCGGACGGAGGGAGTACTTCCTCCTTCCATCTATATAGGGCCTAATGTGTTTTTCAAGA

CAGACTTTGACTATTGACAAGATTAATAGCACATAAGATGTATACTATAAAAATTATAAT

ATTGGAAGCTCCTTTGACATACGAATTTGAAGGTATGCTTTGTGTAAGTTGCATGTCAAA

TATTGTTACTCGAACGTTTGGTCAAAGTTAGCCTCGAAAAACGCATTAGGCCCTATATAG

ATGGAAGAAGGGAGTAAAATGTTCTCCCATAAGAAATTTCCTCATTTTGTATATACCACC

AGAATAATAACAAAATGCAGAGTAAGAAAGATGAAGAAAAACAATAATGCGTTGCACGAT

GCAAAACCAGCAAGGAACACTTAGCAATATATAAACTAATCAATAATGCATGGAGATGGC

AAGGCGGTCAGGATCAGATGAGTCATCGCAACAGCCCAAAGGAAAAGGAAAAGATGGAAT

AGAATACATACATGTGATGATACACCATGCAACATGCTACTGGTCTACCGCGGCGCCCCG

CTACTCTACGCTCCTCACTCTCAGCTCGGCTCAGCTTCTTGGCAGCCTATAAATAGATGC

TCTTGTGCAACTCATCCGGCACACCCATCCTCTTTCAGAAGCACAGAGAGAGATCTTCTA

GCTACACACTGTTGCCGTCGATCCAGCAAAAATG

**b. *TaASN1* gene promoter from TRIAE_CS42_5BL_TGACv1_404794_AA1310950. The ATG translation start site is highlighted in yellow, the putative TATA box is underlined and the putative N-motif is highlighted in red.**

GACCCCGGCGTCTGCTTCGTAACCCAAACGCCAGGGACCTTAGCGTCACCGAAAAAGGTC

AGAAAGAAAAAAAATTCGGAACGAGGTCAAATCGGGATTTAGTTTGCCTTAAGGGTCAGA

ACAGTAATCCCTCCCCAGAGTGGATACAAGTTTTTGCAAGGACTGGGCACGTAGCAATAG

AGCCAAGAGCAGCTATTGCTAGCCACAGTCACGCACATGAACCGCTCATGTCGACACTCG

TACCAATAATGCCGGACGGAGGACGAACATGTCCTCCTGCTACGTAAGCGATGGGCAGCA

TAGCATATTCATTCAAGGAGATCCGGATCACGCAACAAGATGCACCCCTGACATACATAT

ACCAAATAAAATGTTCAACAATGCCACCAGAATAATAACCAAATGCAGAGTAAGATAGAT

GAAGAAAAACAATAATGAGTACAGTAGGATGCAAAACCAGCAAGGAACAGGAAGCAATAA

ACTAATCAATAATGCATTGAGATGGCAAGGCGGTCAGGATCAGATGAGTCATCGCAACAG

CCCAAAGGAAAAGGAAAAGATGGAATAGAATACATGTGATAATACACCATGCAACATGCT

ACTGGTCTACCGCGGCGCCCCGCTACGCTCCTCACTCTCAGCTCAGCTCAGCTCTTGGCA

GCCTATAAATAGACGCTCTTGTGCAACTCATACGGCACACCCATCCTCCTTCAGAAGCAC

AGAGAGAGATCTTCTAGCTACATACTGTTGCCGTCGATCCAGCGAAAATG

**c. *TaASN1* gene promoter from TRIAE_CS42_5DL_TGACv1_438333_AA1468150. The ATG translation start site is highlighted in yellow, the putative TATA box is underlined and the putative N-motif is highlighted in red.**

TAGCAATGAGCCCGAATGAGTAGCGGTGGCACATGCAGCTCCAGAGTGCAGAGTGGATAC

AAGTTTTTGCAAGGACTGGGCACGTAGCAATAGAGCCAAGAGCAGCTATTGCTAGCCACA

GTCACGCACATGAACGTCTCATGTCGACACTCGTACCAATAATGCCGTTCGTCCTCCTAC

TACGTAAGCGATGGGCAGCATAGCATATTCATTCAAGGAGATCCGGATCACGCAACAAGA

TGCATCCCTGACATACATATACCAAATAAAGTGTTCAACAATACCACCAGAATAATAACC

AAATGCAGAGTAAGATACACGAAGAAAAACAATAATGAGTAGGATGCAAAACCAGCAAGG

AACACGAACCAATAAACTACTCCTTCCGTTCCTAAATACTTGTCTTTCTAGGCATTTCAA

CAAATGACTACATACAGAGTAAAATGAGTGAATCTACACTCTAAAATATGTCTACATACA

TCCGTATGTGATATTCATTTGAAATGCCTAGAAAGACAAGTATTTAGGAACGGAGGGAGT

AATCAATAATGCATGGAGATGGCAAGGCGGTCAGGATCAGATGAGTCATCGCAACAGCCC

AAAGGAAAAGGAAAAGATGGAATAGAATACATGTGATAATACACCATGCAACATGCTACT

GGTCTACCGCGGCGCCCCGCTACGCTCCTCACTCTCAGCTCAGCTCAGCTCTTGGCAGCC

TATAAATAGACGCTCTTGTGCAACTCATACGGCACACCCATCCTCCTTCAGAAGCACAGA

GAGAGATCTTCTAGCTACATACTGTTGCCGTCGATCCAGGAAAATG

**d. *TaASN2* gene promoter from TRIAE_CS42_3DS_TGACv1_271746_AA0907290.** **The ATG translation start site is highlighted in yellow, the putative TATA box is underlined and the adjacent C-box is highlighted in red.**

-721 CACAAACACCCTAAACCCTAC

-700 ATACCTCTATGAGCACCTTCGAGAAACCGAGCCGACACATCATCTTGAAA

-650 TTCACAAAGTCATCATAGACGTCTTTATATTTGACGGGTATGTCCCCTCC

-600 CGCTGAACGCACGTCGCCGGAAGACCAGGGATAAAAATCTAATAAAATGC

-550 AAGCATTAATGTTAAGTTTAGAACTTAAACTCTAGTAGGCTGGAAATACA

-500 CTGTCCACCTAACCGTCCAACTATAGGTTGATTGGTTCTACCTTCCATTT

-450 ACTTGTAGGTTCAATGTGTGTGTGTGGGTGGGGGTGGGGGGCATAGTCGG

-400 AGCCTGAAGAAGAGGGAAAAAACGTCGAGGATTGACTCGATACTACTAGC

-350 TGGTAATGGATGATGCGAGAAGAGAAAGACGTGGAGTACATGCATGGACA

-300 TGTAACAGGCAGAAACGCATGCATGCACGATGCTGATGGATGATGGCAGA

-250 AGTGGACAAACGAACCCATGGTCTGATGTGGTGCGGTGGCCGCGAGAGAA

-200 TCCGACGAGGCCGTGCATGGAGGACGTGTAGCTCCGCTCTCCGCCATCTC

-150 CGGCCAGGCATCTATCTACCTACAAGTAGAGCCAAGCCATTCCTGCACAC

-100 CTCCATACACAAACACAATTCAGACCGACTACCTCGCTGCTCGCTGTAGA

-50 CGACGTTCGACGACGATCCAGAGGAGCAGCATAACCGAGGAGAGCGGAGCATG

**Table S1**

Transcription factors differentially expressed in response to sulphur deficiency, either up or down, or up/down exclusively.

| TF family | SR3 UP | Spark UP | SR3 DOWN | SPARK DOWN | Spark UP/SR3 DOWN |
| --- | --- | --- | --- | --- | --- |
| bZIP | 5 | 14 | 8 | 4 | 2 |
| CPP | 2 | 0 | 0 | 3 | 0 |
| ARF | 1 | 1 | 2 | 7 | 0 |
| ERF | 14 | 12 | 45 | 3 | 0 |
| HD-ZIP | 1 | 2 | 6 | 8 | 0 |
| bHLH | 5 | 7 | 22 | 12 | 0 |
| AP2 | 1 | 2 | 2 | 3 | 0 |
| WOX | 0 | 2 | 0 | 3 | 0 |
| Dof | 3 | 0 | 6 | 1 | 0 |
| HRT-like | 0 | 0 | 0 | 1 | 0 |
| NAC | 11 | 11 | 22 | 1 | 0 |
| B3 | 3 | 1 | 5 | 6 | 0 |
| GRAS | 4 | 3 | 12 | 3 | 0 |
| C3H | 0 | 1 | 2 | 1 | 0 |
| GRF | 0 | 0 | 1 | 5 | 0 |
| HSF | 1 | 0 | 28 | 5 | 0 |
| DBB | 0 | 0 | 4 | 2 | 0 |
| Trihelix | 8 | 2 | 4 | 2 | 0 |
| G2-like | 2 | 0 | 6 | 2 | 0 |
| WRKY | 3 | 2 | 43 | 2 | 0 |
| MYB | 10 | 5 | 13 | 2 | 0 |
| NF-YA | 1 | 0 | 0 | 1 | 0 |
| SRS | 0 | 0 | 0 | 1 | 0 |
| C2H2 | 5 | 2 | 12 | 1 | 0 |
| NF-YB | 5 | 0 | 3 | 0 | 0 |
| SBP | 0 | 3 | 1 | 0 | 0 |
| MYB_related | 7 | 1 | 13 | 0 | 0 |
| FAR1 | 11 | 4 | 3 | 0 | 0 |
| GATA | 4 | 1 | 2 | 0 | 0 |
| EIL | 0 | 3 | 4 | 0 | 0 |
| LBD | 3 | 0 | 4 | 0 | 0 |
| CAMTA | 0 | 0 | 1 | 0 | 0 |
| M-type | 0 | 0 | 1 | 0 | 0 |
| MIKC | 2 | 1 | 2 | 0 | 0 |
| CO-like | 2 | 0 | 3 | 0 | 0 |
| NF-X1 | 0 | 0 | 3 | 0 | 0 |
| E2F/DP | 5 | 1 | 0 | 0 | 0 |
| GeBP | 0 | 2 | 0 | 0 | 0 |
| HB-other | 1 | 2 | 0 | 0 | 0 |
| ARR-B | 1 | 1 | 0 | 0 | 0 |
| HB-PHD | 1 | 0 | 0 | 0 | 0 |
| S1Fa-like | 3 | 0 | 0 | 0 | 0 |
| ZF-HD | 2 | 0 | 0 | 0 | 0 |
| Total | 127 | 86 | 283 | 79 | 2 |
